# LncRNA WEE2-AS1 is a diagnostic biomarker that predicts poor prognoses in patients with glioma

**DOI:** 10.1186/s12885-023-10594-y

**Published:** 2023-02-06

**Authors:** Xuqiang Zhu, Di Chen, Yiyu Sun, Shuo Yang, Weiguang Wang, Bing Liu, Peng Gao, Xueyuan Li, Lixin Wu, Siqi Ma, Wenyang Lin, Jiwei Ma, Dongming Yan

**Affiliations:** 1 Department of Neurosurgery, The First Affiliated Hospital of Zhengzhou University, 450052 Zhengzhou, Henan China; 2 Department of Neurosurgery, School of Medicine, Shanghai Jiao Tong University Affiliated Sixth People’s Hospital, Shanghai Jiao Tong University, 200030 Shanghai, China; 3 Department of Anatomy and Physiology, Shanghai Jiao Tong University School of Medicine, 200025 Shanghai, China; 4 Department of Neurosurgery, The First Affiliated Hospital of Xinxiang Medical University, Xinxiang, 453100 Henan Shanghai, China

**Keywords:** WEE2-AS1, Glioma, Biomarker, Survival, Prognosis, RNA-Seq

## Abstract

**Background:**

Glioma is characterized by high morbidity, high mortality, and poor prognosis. Despite tremendous advances in the treatment of glioma, the prognosis of patients with glioma is still unsatisfactory. There is an urgent need to discover novel molecular markers that effectively predict prognosis in patients with glioma. The investigation of the role of WEE2-AS1 in various tumors is an emerging research field, but the biological function and prognostic value of WEE2-AS1 in glioma have rarely been reported. This study aimed to assess the value of WEE2-AS1 as a potential prognostic marker of glioma.

**Methods:**

Gene expression (RNA-Seq) data of patients with glioma were extracted from The Cancer Genome Atlas (TCGA) and the Genotype-Tissue Expression (GTEx) databases. The Wilcoxon rank sum test was used to analyze the expression of WEE2-AS1 in the cells and tissues of glioma. The Kruskal–Wallis rank sum test, Wilcoxon rank sum test, and logistic regression were used to evaluate the relationship between clinical variables and expression of WEE2-AS1. Cox regression analysis and the Kaplan–Meier method were used to evaluate the prognostic factors in glioma. A nomogram based on Cox multivariate analysis was used to predict the impact of WEE2-AS1 on glioma prognosis. Gene Set Enrichment Analysis (GSEA) was used to identify key WEE2-AS1-associated signaling pathways. Spearman’s rank correlation was used to elucidate the association between WEE2-AS1 expression and immune cell infiltration levels.

**Results:**

We found that WEE2-AS1 was overexpressed in a variety of cancers, including glioma. High expression of WEE2-AS1 was associated with glioma progression. We determined that the expression of WEE2-AS1 might be an independent risk factor for the survival and prognosis of patients with glioma. We further observed that the mechanism of WEE2-AS1-mediated tumorigenesis involved neuroactive ligand-receptor interaction, cell cycle, and the infiltration of immune cells into the glioma microenvironment.

**Conclusion:**

These findings demonstrate that WEE2-AS1 is a promising biomarker for the diagnosis and prognosis of patients with glioma. An increased understanding of its effects on the regulation of cell growth may lead to the development of clinical applications that improve the prognostic status of patients with glioma.

**Supplementary Information:**

The online version contains supplementary material available at 10.1186/s12885-023-10594-y.

## Introduction

Glioma is the most common primary malignant tumor originating from glial cells [1, 2]. Currently, the standard treatment to reduce the rate of mortality in patients with glioma is maximum safe surgical resection, followed by postoperative radiotherapy and chemotherapy [3–5]. With continuous improvement at the medical level, new treatment methods, such as alternating electric field therapy, targeted therapy, and immunotherapy, have been proposed [6]. However, the prognosis of patients with glioma fails to satisfy the expected goal and the median survival time is only 12–18 months [1, 7, 8]. The main factors contributing to the poor prognoses are the heterogeneity of tumors and the complexity of epigenetics, making it difficult to identify the therapeutic target of gliomas [3, 9]. In addition, the infiltrative nature of tumors makes it practically impossible to completely remove the tumor by surgical procedures, and the existence of the physiological blood-brain barrier limits the effect of drugs [1]. Thus, an in-depth exploration of the molecular mechanisms of tumor occurrence and progression is essential for the improvement of clinical diagnosis and treatment and the discovery of novel and effective molecular markers.

With the rapid development of molecular pathology in recent years, some molecular markers have been proven to play a valuable role in improving diagnostic accuracy and prognostic stratification of glioma [10]. For instance, isocitrate dehydrogenase 1 or 2 (IDH1 or IDH2) mutations and 1p/19q codeletion confer a favorable prognosis in patients treated with radiation or alkylating chemotherapy [6, 11, 12]. Patients with methylation of the promoter region of O^6^-methylguanine-DNA methyltransferase acquire more benefits from treatment with temozolomide than those lacking methylation promoters [6, 13]. Although these molecular markers have been used for the clinical diagnosis and treatment of glioma, the tumorigenesis of glioma is a very complex pathological process involving a variety of molecules [10]. Therefore, the search for additional molecular markers is crucial in the diagnosis, treatment, and prevention of glioma.

LncRNA WEE2 antisense RNA1 (WEE2-AS1) is the antisense RNA of the gene encoding WEE1 homolog 2 [14]. Recent studies suggest that WEE2-AS1 plays a key role in the development, metastasis, and invasion of various tumors [15–17]. For instance, WEE2-AS1 accelerates the growth and progress of hepatocellular carcinoma [15]. Similarly, WEE2-AS1 promotes proliferation and inhibits apoptosis in triple negative breast cancer cells via regulating the miR-32-5p/TOB1 axis [16]. Moreover, WEE2-AS1 has been reported to participate in the growth and progression of glioma [17, 18], but the role of WEE2-AS1 as a potential glioma diagnostic and prognostic marker has not been fully elucidated.

In this study, we explored the association between WEE2-AS1 and glioma, especially with respect to clinicopathological features of glioma, risk factors for reduced survival, and the involvement of specific molecular pathways. For all we know, our finding provides powerful evidence that WEE2-AS1 is a promising biomarker for the diagnosis and prognosis of patients with glioma, which can improve the prognostic status of patients with glioma.

## Materials and methods

### Data acquisition and processing

The RNA-Seq data of Cancer Genome Atlas (TCGA) and Genotype-Tissue Expression (GTEx) were downloaded in transcripts per kilobase million (TPM) format from the UCSC Xena database (http://xenabrowser.net/datapages/) and uniformly processed with the Toil process [19]. The data of 1157 normal brain tissues are from the GTEx (1152 normal brain tissues) and TCGA (5 para-cancer tissues) databases (https://cancergenome.nih.gov/). 670 glioma tissue data including glioblastoma (GBM) and low-grade glioma (LGG), and clinical information were collected from TCGA database (https://cancergenome.nih.gov/) [20]. At the same time, we further screened 325 glioma patients with complete clinical information from Chinese glioma genome atlas (CGGA, http://www.cgga.org.cn/) database, and analyzed the expression level of WEE2-AS1 and overall survival in the patients with glioma. These databases are publicly open-access and available and therefore there was no need to get approval from the local ethics committee.

### Cell culture

Human glioma cell lines (T98, A172, and U251) and normal control cells (normal human astrocytes, NHAs) were provided by National Collection of Authenticated Cell Cultures (Shang Hai, China). All the above cell lines were cultured in Dulbecco’s Modified Eagle Medium (DMEM; Gibco) medium containing 10% fetal bovine serum (FBS; Gibco) and 1% penicillin–streptomycin at 37 ℃ in air containing 5% CO_2_.

### Tumor samples collection

A total of 10 glioma tissue samples (3 WHO grade II, 3 WHO grade III, and 4 GBM) and 6 corresponding normal brain tissues were obtained from patients who underwent surgical resection between October 2021 and March 2022 at the First Affiliated Hospital of Zhengzhou University, Henan, China. The clinicopathological characteristics of the patients with glioma were summarized in supplementary table S1. The samples were immediately frozen in liquid nitrogen after tissue resection and mRNA expression level was measured using quantitative reverse transcription polymerase chain reaction (RT-qPCR) [10]. This study conforms to the guidelines issued in the Declaration of Helsinki and was approved by the Ethics Committee of the First Affiliated Hospital of Zhengzhou University (Approval Number: 2019-KY-176).

### RNA isolation and RT‑qPCR

The total RNA of the above cell line and tissue samples was extracted using TRIzol reagent (Invitrogen) according to the manufacturer’s protocol. The RNA samples were reverse-transcribed into cDNA by using iScriptTM cDNA Synthesis Kit (BioRad, 1,708,891). RT-qPCR was performed using a thermal cycler (Roche LightCycler 480) using IQTM SYBR® Green Supermixes for Real-Time PCR (Biorad, 1,708,880). The following conditions were used for PCR: 1 cycle of 95 °C for 3 min, followed by 39 cycles of a three-step cycling program (95 °C for 20 s; 60 °C for 20 s; 72 °C for 20 s). The mRNA expression was normalized to the expression of glyceraldehyde-3-phosphate dehydrogenase (GAPDH) mRNA and calculated by the 2^−ΔΔCt^ method. Specific primers for WEE2-AS1 and GAPDH were as follows: WEE2-AS1 forward 5′-ATGGGGCTGTACTGAATACCG-3′and reverse 5′-ATACCATGATCACGAAGGTGGTT-3′, and GAPDH forward 5′-TGTGGGCATCAATGGATTTGG-3′ and reverse 5′-ACACCATGTATTCCGGGTCAAT-3′.

### Identification of differentially expressed genes (DEGs) and functional enrichment analysis

670 glioma patients were divided into high- and low-expression groups according to the WEE2-AS1 expression (median ± upper and lower interquartile range) from TCGA. DEGs were analyzed using the DESeq2 package on HTSeq-counts data [21]. |log2fold-change (FC)| > 2 and adjusted p < 0.05 were considered threshold values for DEGs. Gene Ontology (GO) functional analyses, including Cellular Component (CC), Molecular Function (MF), Biological Processes (BP), and Kyoto Encyclopedia of Genes and Genomes (KEGG) pathway enrichment analyses, were performed on DEGs using the clusterProfiler package [22] in R (version 3.6.2) with p values adjusted using the Benjamini and Hochberg method. Besides, we get permission to use the KEGG software from the Kanehisa laboratory [23–25].

### Gene set enrichment analysis (GSEA)

GSEA was performed using the clusterProfiler package to elucidate the significant function and pathway differences between high- and low-expression WEE2-AS1 groups [22, 26]. The c2.all.v7.0.symbols.gmt [Curated] in the MSigDB Collections was selected as the reference gene set. The number of gene set permutations was 1000 for each analysis. The pathway enrichment was analyzed based on a false discovery rate (FDR) < 0.25 and adjusted p < 0.05.

### Analysis of immune infiltration

Data on the marker gene of 24 immune cell types were obtained from an immunity study [27], and the infiltration of 24 immune cell types within the 670 glioma samples from TCGA (GBM-LGG) was analyzed by single-sample GSEA (ssGSEA) using the GSVA package [28]. The 24 types of immune cells in the glioma included dendritic cells (DCs), mast cells, natural killer CD56 bright cells, T follicular helper (Tfh) cells, plasmacytoid dendritic cells (pDCs), CD8 T cells, Th1 cells, Tregs, effector memory T cells (Tems), natural killer CD56dim cells, central memory CD4 + T cells (Tcms), B cells, cytotoxic cells, natural kill (NK) cells, Th17 cells, T cells, γδ T cells (Tgds), immature DCs (iDCs), T helper cells, eosinophils, neutrophils, activated DCs (aDCs), Th2 cells, and macrophages. Additionally, the correlations between WEE2-AS1 and these 24 cell types were analyzed using Spearman’s rank correlation.

### Prognostic prediction model building and evaluation

Multivariate Cox regression analysis was used to determine the optimal model and a nomogram was constructed to predict the prognosis of patients with glioma using the rms package (version 5.1-3; http://cran.rproject.org/w-eb/packages/rms/index.html) [29]. The C-index was used to evaluate the pretesting capability of the module — the C-index between 0.50 and 0.70 was of low accuracy, between 0.71 and 0.90 was of medium accuracy; and above 0.90 was of high accuracy. The calibration plot was a comparison between the premeasured risk and the actual risk of the patient used to determine the accuracy of the nomogram.

### Statistical analysis

All statistical data and plots were generated using the R program (version 3.6.3). The Wilcoxon rank sum test was used to analyze the expression of WEE2-AS1 in the cells and tissues of glioma. The Kruskal–Wallis rank sum test, Wilcoxon rank sum test, and logistic regression were used to evaluate the relationship between clinical variables and the expression of WEE2-AS1. Kaplan-Meier methods were used to evaluate the prognostic value of WEE2-AS1 in overall survival (OS, the time from randomization until death from any cause), progression-free survival (PFS, the time from randomization until the date of objective disease progression or death from any cause in the absence of progression), and disease-specific survival (DSS, the percentage of people in a study or treatment group who have not died from a specific disease in a defined period of time) of gliomas. Univariate and multivariate cox regression analyses were used to evaluate prognostic factors, with univariate analysis of variables as p < 0.2 considered for multivariate analysis. The data in the graphs represent the median ± upper and lower interquartile range. The receiver operating characteristic (ROC) curve was used to quantitatively evaluate the diagnostic value of WEE2-AS1 in glioma by using the data of GTEx and TCGA and the pROC package [30]. An area under the curve (AUC) value between 0.5 and 0.7 was of low accuracy; between 0.7 and 0.9, of medium accuracy; and above 0.9, of high accuracy.

## Results

### Clinical characteristics of patients with glioma

Table 1 shows the clinical information from TCGA (LGG-GBM) of 670 patients with glioma, whose data were used in this study, including age, gender, race, primary therapy outcome, WHO grade, histological type, IDH status, 1p/19q codeletion, epidermal growth factor receptor (EGFR) status and phosphatidylinositol-4,5-bisphosphate 3-kinase catalytic subunit alpha (PIK3CA) status. Moreover, the Chi-square test or Fisher’s exact test found that the expression of WEE2-AS1 was significantly associated with WHO grade (p < 0.001), histological type (p < 0.001), IDH status (p < 0.001), 1p/19q codeletion (p < 0.001), and EGFR status (p = 0.004). A *t*-test or Wilcoxon rank sum test determined that WEE2-AS1 was significantly correlated with age (p = 0.005). The clinicopathological characteristics of 325 patients with glioma from CGGA were summarized in supplementary table S2.

**Table 1:** Association between LncRNA WEE2-AS1 expression and clinicopathological features in the validation cohort

Characters	Level	Low expression of WEE2-AS1	High expression of WEE2-AS1	*p*	Test
N		335	335		
Age (%)	<=60	274(81.8%)	257(76.7%)	0.127	exact
	> 60	61(18.2%)	78(23.3%)		
Gender (%)	Female	141(42.1%)	143(42.7%)	0.938	exact
	Male	194(57.9%)	192(57.3%)		
Race (%)	Asian	5(1.5%)	8(2.4%)	0.643	exact
	Black or African American	15(4.6%)	17(5.2%)		
	White	309(93.9%)	304(92.4%)		
Primary therapy outcome (%)	CR	78(31.6%)	57(28.9%)	0.072	exact
	PD	46(18.6%)	57(28.9%)		
	PR	35(14.2%)	27(13.7%)		
	SD	88(35.6%)	56(28.4%)		
WHO grade (%)	G2	130(42.5%)	86(28.0%)	< 0.001	exact
	G3	127(41.5%)	110(35.8%)		
	G4	49(16.0%)	111(36.2%)		
Histological type (%)	Astrocytoma	93(27.8%)	99(29.6%)	< 0.001	exact
	Oligodendroglioma	132(39.4%)	58(17.3%)		
	Oligoastrocytoma	61(18.2%)	67(20.0%)		
	Glioblastoma	49(14.6%)	111(33.1%)		
IDH status (%)	Mut	247(74.4%)	177(53.8%)	< 0.001	exact
	WT	85(25.6%)	152(46.2%)		
1p/19q codeletion (%)	Codel	127(37.9%)	41(12.5%)	< 0.001	exact
	Non-codel	208(62.1%)	288(87.5%)		
EGFR status (%)	Mut	25(7.6%)	48(14.7%)	0.004	exact
	WT	304(92.4%)	279(85.3%)		
PIK3CA status (%)	Mut	26(7.9%)	23(7.0%)	0.767	exact
	WT	303(92.1%)	304(93.0%)		
Age (median [IQR])		44.00[33.00,56.00]	48.00[35.00,59.50]	0.005	non-norm

Column 6 in the table: The chi-square test is used by default for categorical variables, and ‘exact’ indicates that the statistical method is Fisher’s exact test. Numerical variables are statistically analyzed using t.test by default. “non-norm” was expressed as a non-normal distribution, and statistical analysis was performed using the Wilcoxon rank sum test

### High expression of WEE2-AS1 in gliomas cells and tissues

**Fig. 1:**
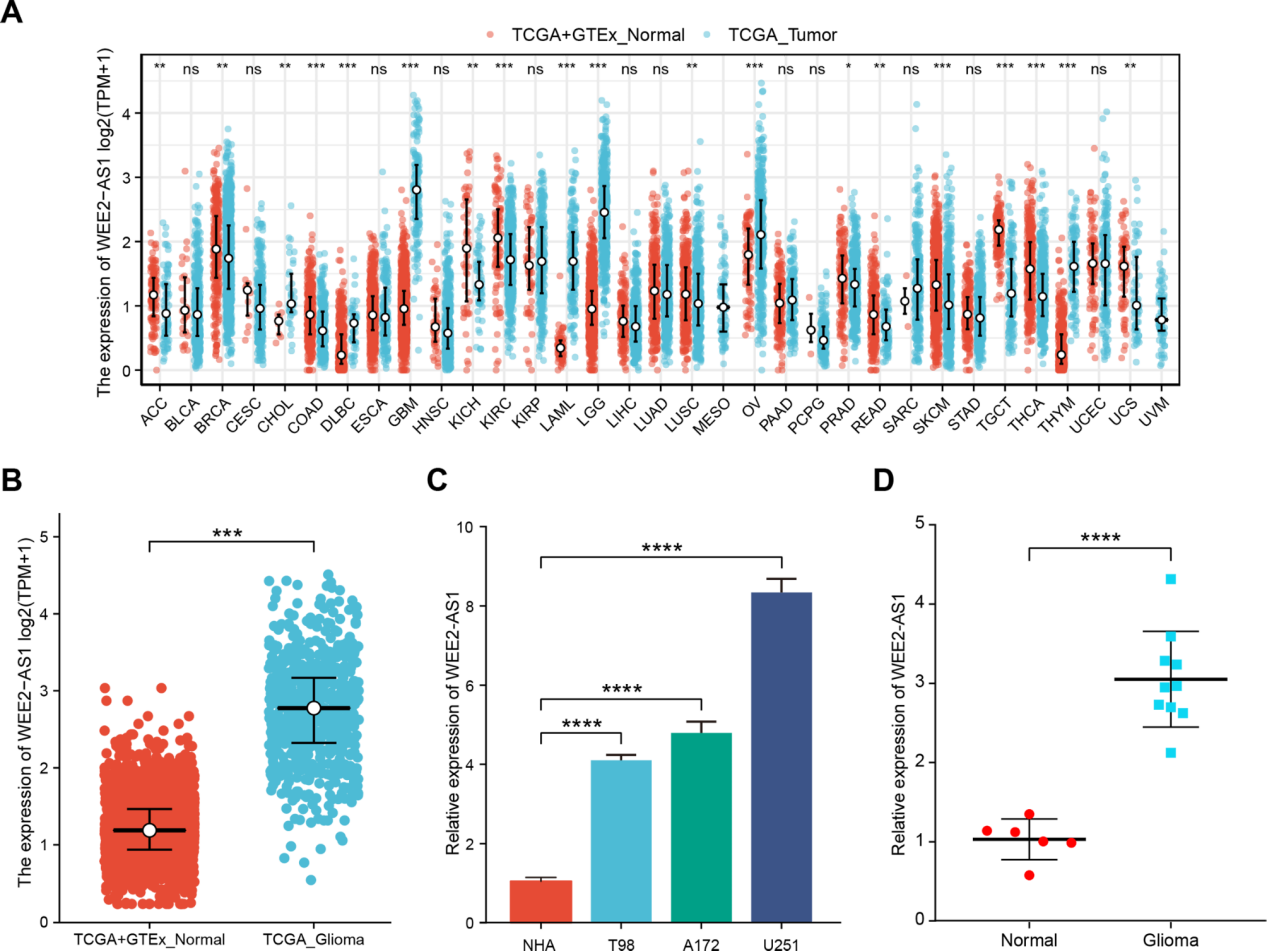
WEE2-AS1 expression in different types of tumors, including glioma (A) and (B) The expression levels of WEE2-AS1 in 33 tumor tissues (A) and glioma tissues (B) from RNA-Seq data in The Cancer Genome Atlas (TCGA) and the Genotype-Tissue Expression (GTEx) databases (Wilcoxon rank sum test, p < 0.001) (ACC, adrenal cortical carcinoma; BLCA, bladder urothelial carcinoma; BRCA, breast invasive carcinoma; CESC, Cervical squamous cell carcinoma and endocervical adenocarcinoma; CHOL, cholangiocarcinoma; COAD, colon adenocarcinoma; DLBC, lymphoid neoplasm diffuse large b-cell lymphoma; ESCA, esophageal carcinoma; GBM, glioblastoma; HNSC, head and neck squamous cell carcinoma; KICH, kidney chromophobe; KIRC, kidney renal clear cell carcinoma; KIRP, kidney renal papillary cell carcinoma; LAML, acute myeloid leukemia; LGG, lower grade glioma; LIHC, liver hepatocellular carcinoma; LUAD, lung adenocarcinoma; LUSC, lung squamous cell carcinoma; MESO, mesothelioma; OV, ovarian serous cystadenocarcinoma; PAAD, pancreatic adenocarcinoma; PCPG, pheochromocytoma and paraganglioma; PRAD, prostate adenocarcinoma; READ, rectum adenocarcinoma; SARC, sarcoma; SKCM, skin cutaneous melanoma; STAD, stomach adenocarcinoma; TGCT, testicular germ cell tumors; THCA, thyroid carcinoma; THYM, thymoma; UCEC, uterine corpus endometrial carcinoma; UCS, uterine carcinosarcoma; UVM, uveal melanoma). (C) Quantitative real-time polymerase chain reaction (RT-qPCR) analysis was used to detect WEE2-AS1 expression in three glioma cell lines (T98, U251, and A172) and a normal human astrocyte (NHA) cell line (unpaired *t*-test, p < 0.0001). (D) WEE2-AS1 expression in 10 glioma tissues and 6 normal brain tissues was determined using RT-qPCR (unpaired *t*-test, p < 0.0001). Data are represented as median ± upper and lower interquartile range. ns, no significance; *p < 0.05, **p < 0.01, ***p < 0.001, ****p < 0.0001

To investigate the expression of WEE2-AS1 in normal tissues and tumors, the downloaded RNA-Seq data in TPM format from TCGA and GTEx was processed uniformly using the TOIL Xena (https://xenabrowser.net/datapages/) process from the University of California, Santa Cruz [19]. As seen in Fig. 1A and table S3, the expression of WEE2-AS1 in cholangiocarcinoma (CHOL), diffuse large B-cell lymphoma (DLBC), Glioblastoma (GBM), acute myeloid leukemia (LAML), lower-grade glioma (LGG), ovarian cancer (OV), and thymoma (THYM) was higher than that in normal tissues (P < 0.05).

We then analyzed RNA-Seq data of normal tissues (n = 1157) and glioma tissues (LGG-GBM, n = 670) from the GTEx and TCGA databases [19, 20] and found that WEE2-AS1 expression was also significantly higher in human glioma tissues than in normal tissues (p < 0.001) (Fig. 1B). These results also apply to the 325 glioma tissues and 20 normal tissues from CCGA databases (Fig. S1A). Additionally, we further validated that WEE2-AS1 levels were upregulated in glioma cells (T98, U251, and A172) compared with NHA cells using RT-qPCR (Fig. 1C). We also investigated the expression of WEE2-AS1 in 10 cases of glioma and 6 normal brain tissues. The results of RT-qPCR suggest that WEE2-AS1 was elevated in gliomas compared with the corresponding normal brain tissues (Fig. 1D).

Collectively, these results revealed that WEE2-AS1 is consistently upregulated in both glioma cells and tissues compared with corresponding normal cells and tissues.

### Correlations between WEE2-AS1 and clinical features of glioma

**Fig. 2:**
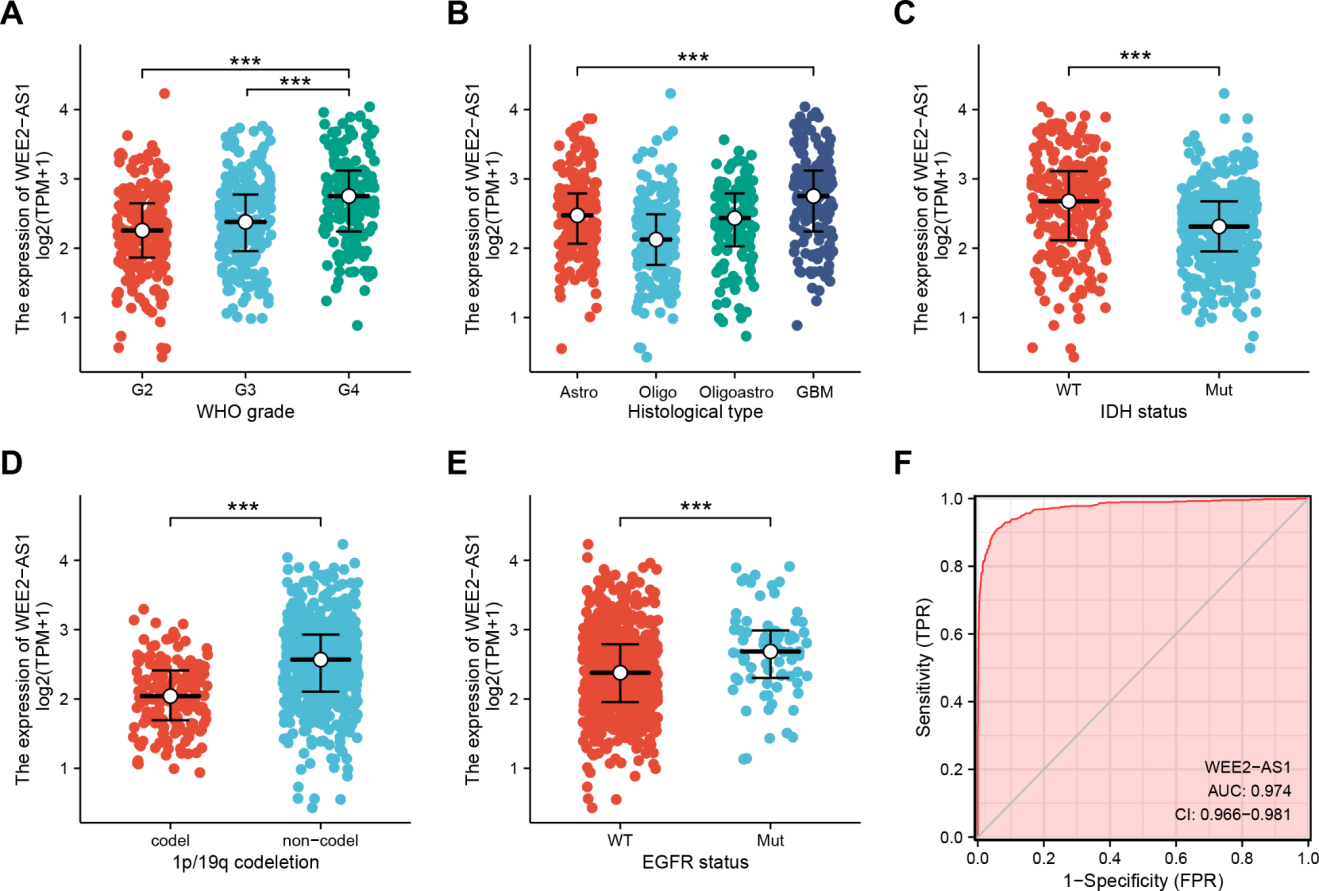
Correlations between WEE2-AS1 and clinical features of glioma (A)–(E) The relationship between the WEE2-AS1 expression and the WHO grade (A), histological type (Astro, Astrocytoma; Oligo, Oligodendroglioma; Oligoastro, Oligoastrocytoma; GBM, Glioblastoma) (B), isocitrate dehydrogenase (IDH) status (C), 1p/19q codeletion status (D), and epidermal growth factor receptor (EGFR) status (E) of glioma by using the data of TCGA (Wilcoxon rank sum test, p < 0.001). (F) Receiver operating characteristic (ROC) analysis of WEE2-AS1 expression shows a high ability to discriminate glioma tissues from normal samples using the data of GTEx and TCGA. Data are represented as median ± upper and lower interquartile range. ****p < 0.0001

To clarify the correlation between the expression of WEE2-AS1 and the clinical, pathological, and molecular characteristics of patients with glioma, we analyzed the relationship between the WEE2-AS1 expression and the WHO grade, histological type, IDH status, 1p/19q codeletion status, and EGFR status of glioma, by using the data of TCGA (LGG-GBM). As shown in Fig. 2A and Fig. S2A, the expression of WEE2-AS1 was significantly higher in WHO grade IV gliomas than in WHO grade II and III gliomas. Additionally, the expression of WEE2-AS1 in glioblastoma was higher than that in astrocytoma, oligodendroglioma, and oligoastrocytoma (Fig. 2B). Previous studies found that IDH, 1p/19q, and EGFR are recognized molecular markers associated with the survival and prognosis of gliomas [6, 31]. Our findings revealed that the expression of WEE2-AS1 was higher in patients without 1p/19q codeletion, patients with wild-type (WT) IDH, and patients with Mut-type EGFR (Fig. 2C–E). While, no difference in WEE2-AS1 expression was observed in MGMT promoter methylated and unmethylated patients from the CGGA databases (Fig. S1C). Overall, the results indicated that the increased expression of WEE2-AS1 was associated with various malignancies of glioma.

In addition, the correlation between WEE2-AS1 expression and major clinicopathological factors, including age (≤ 60 vs. >60, p = 0.01), WHO grade (G2 vs. G3 and G4, p < 0.001), histological type (astrocytoma, oligoastrocytoma, oligodendroglioma vs. glioblastoma, p < 0.001), IDH status (WT vs. Mut, p < 0.001), 1p/19q codeletion status (Codel vs. Non-codel, p < 0.001), and EGFR (WT vs. Mut, p < 0.001) were further determined by using logistic regression, as shown in Table 2. The results indicated that the expression of WEE2-AS1 is associated with poor clinicopathological factors.

**Table 2:** Association of lncRNA WEE2-AS1 expression with clinical pathological characteristics by logistic regression

Characteristics	Total (N)	Odds Ratio (OR)	*P*
Age ( < = 60 vs. >60)	670	0.92(0.86–0.98)	0.01
Gender (Female vs. Male)	670	0.96(0.90–1.01)	0.141
Race (Asian vs. Black or African American & White)	658	1.08(0.89–1.28)	0.396
Primary therapy outcome (CR vs. PD & SD & PR)	444	0.95(0.87–1.03)	0.245
WHO grade (G2 vs. G3 & G4)	613	0.84(0.78–0.90)	< 0.001
Histological type (Astrocytoma & Oligoastrocytoma & Oligodendroglioma vs. Glioblastoma)	670	0.80(0.75–0.85)	< 0.001
IDH status (WT vs. Mut)	661	1.24(1.17–1.33)	< 0.001
1p/19q codeletion (Codel vs. Non-codel)	664	0.65(0.58–0.72)	< 0.001
EGFR status (WT vs. Mut)	656	0.86(0.79–0.93)	< 0.001
PIK3CA status (WT vs. Mut)	656	1.02(0.92–1.15)	0.703

Furthermore, we evaluated the diagnostic value of WEE2-AS1 in glioma patients by drawing a ROC curve. The results showed that the AUC was 0.974 (95% confidence interval [CI] = 0.966–0.981), suggesting the potential diagnostic role of WEE2-AS1 in glioma (Fig. 2F). In addition, we investigated the correlation between normal samples of high WEE2-AS1 expression and tumor samples of high WEE2-AS1 expression using the available sets of paired normal + tumor glioma samples from TCGA and GTEx. The results showed that the high normal expression of WEE2-AS1 was associated with high glioma expression (r = 1.000, p < 0.001), indicating that normal brain high expression levels of WEE2-AS1 could be a sort of biomarker for increased glioma risk (Fig. S2B).

### High expression of WEE2-AS1 implies a poor prognosis for patients with glioma

**Fig. 3:**
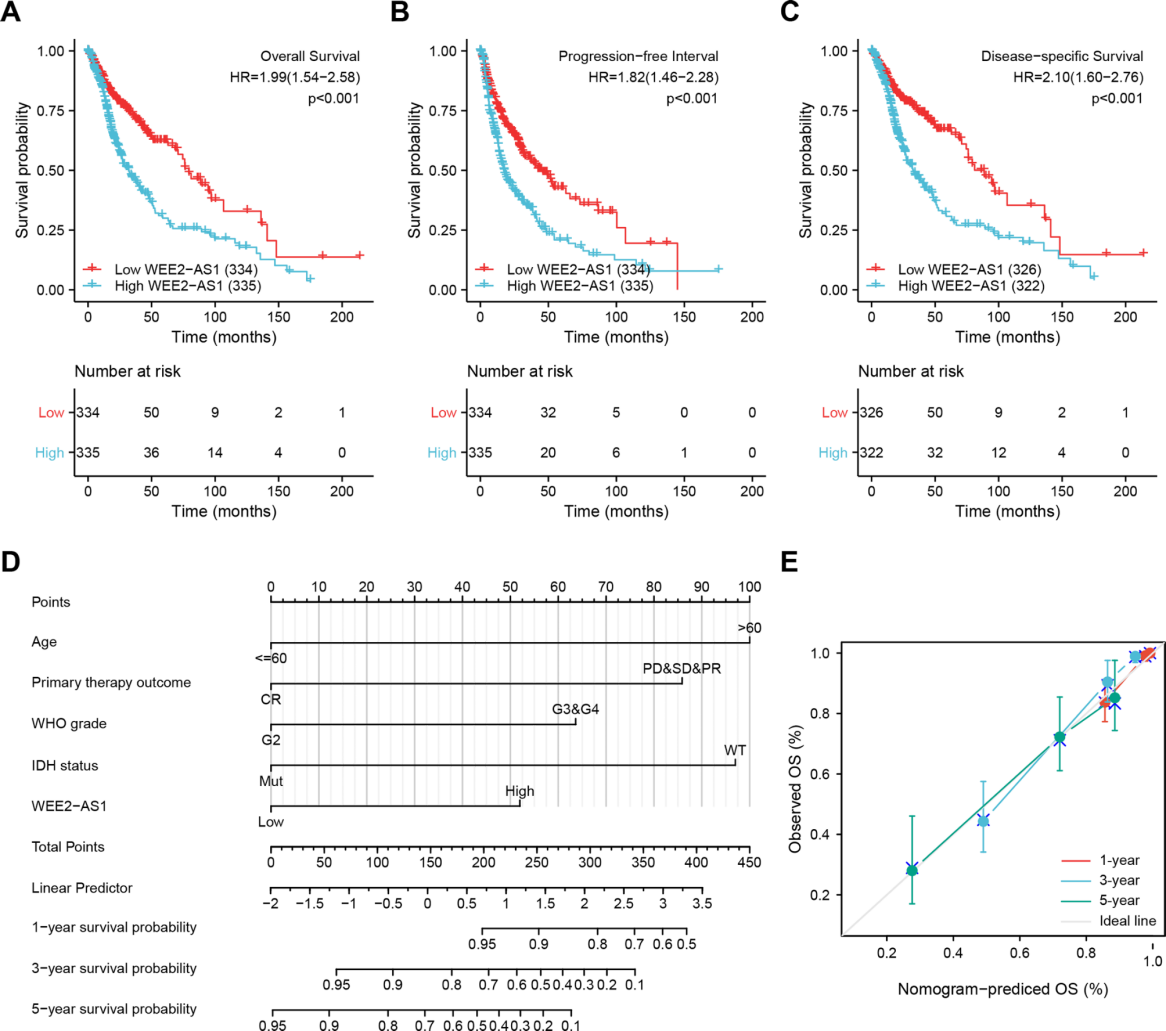
The relationship between WEE2-AS1 and the survival of patients with glioma (A)–(C) Kaplan–Meier survival analysis showing that effect of WEE2-AS1 expression level on overall survival (OS) (A), progression-free interval (PFI) (B), and disease-specific survival (DSS) (C) in patients with glioma in TCGA cohort. (D) Nomogram for predicting the probability of 1-, 3-, and 5-year OS for patients with glioma. (E) Calibration plot of the nomogram for predicting the probability of OS at 1, 3, and 5 years. The abscissa is the probability of the prognosis predicted by the model (0–1 indicates that the probability of the event occurring is 0–100%) and the ordinate is the actually observed prognosis. The colored line is the fitted line indicating the predicted value (the horizontal axis) corresponding to the actual value (the vertical axis). The gray diagonal is the ideal case

To explore whether WEE2-AS1 could be a prognostic factor for glioma, we conducted a Kaplan–Meier survival analysis by using the data of TCGA (LGG-GBM). The results of the survival analysis showed that the patients with high WEE2-AS1 expression had significantly poorer overall survival (OS; hazard ratio [HR] = 1.99, 95% CI, 1.54–2.58, p < 0.001), progression-free interval (PFI; HR = 1.82, 95% CI, 1.46–2.28, p < 0.001) and disease-specific survival (DSS; HR = 2.10, 95% CI, 1.60–2.76, p < 0.001) than those in patients with low WEE2-AS1 expression (Fig. 3A–C). These findings indicated that WEE2-AS1 expression in gliomas is inversely proportional to the survival rate in patients with glioma.

Next, by utilizing the Cox regression model, we computed both univariate and multivariate HRs for patients with glioma. As shown in Table 3, univariate Cox regression analysis showed that the expression of WEE2-AS1 was an independent variable to predict the outcome of OS in patients with glioma (HR = 1.995, 95% CI, 1.544–2.577, p < 0.001). Multiple Cox regression analysis also revealed that WEE2-AS1 expression level was an independent determinant of the prognosis of OS in patients with glioma (HR = 2.002, 95% CI, 1.279–3.133, p = 0.002) after adjusting for age, primary therapy outcome, WHO grade, histological type, and IDH status. Additionally, univariate and multivariate analyses were used to assess the prognostic factors of PFI and DSS with the Cox regression model. The results showed that increased WEE2-AS1 levels were related to reduced PFI and DSS (Table S4 and Table S5).

**Table 3:** Associations between overall survival and clinicopathological characteristics in patients in TCGA using Cox regression

Characteristics	Total (N)	HR (95% CI) Univariate analysis	*P* value Univariate analysis	HR (95% CI) Multivariate analysis	*P* value Multivariate analysis
Age ( < = 60 vs. >60)	669	0.212(0.162–0.277)	< 0.001	0.267(0.161–0.442)	< 0.001
Gender (Female vs. Male)	669	0.813(0.631–1.047)	0.109	0.656(0.421–1.024)	0.064
Race (Asian vs. Black or African American & White)	657	0.836(0.267–2.612)	0.757		
Primary therapy outcome (CR vs. PD & SD & PR)	443	0.238(0.115–0.489)	< 0.001	0.268(0.122–0.592)	0.001
WHO grade (G2 vs. G3 & G4)	612	0.170(0.116–0.249)	< 0.001	0.450(0.275–0.736)	0.001
Histological type (Astrocytoma & Oligoastrocytoma & Oligodendroglioma vs. Glioblastoma)	669	0.111(0.084–0.145)	< 0.001	0.405(0.116–1.413)	0.156
IDH status (WT vs. Mut)	660	9.850(7.428–13.061)	< 0.001	3.409(1.920–6.051)	< 0.001
1p/19q codeletion (Codel vs. Non-codel)	663	0.216(0.138–0.338)	< 0.001	0.891(0.493–1.611)	0.702
EGFR status (WT vs. Mut)	655	0.276(0.203–0.374)	< 0.001	0.654(0.312–1.370)	0.261
PIK3CA status (WT vs. Mut)	655	0.989(0.612–1.601)	0.966		
WEE2-AS1 (High vs. Low)	669	1.995(1.544–2.577)	< 0.001	2.151(1.370–3.378)	< 0.001

Based on the results of multivariate analysis, we conducted a further analysis by establishing the nomogram model to predict the rates of OS, PFI, and DSS for glioma patients 1, 3, and 5 year(s) after surgery. The concordance indexes (C-indexes) of the OS, PFI, and DSS were 0.856 (95% CI, 0.837–0.874), 0.745 (95% CI, 0.725–0.764), and 0.864 (95% CI, 0.846–0.882), respectively (Fig. 3D, Fig. S3A and S3C). In addition, we further developed calibration curves to verify the effectiveness of nomogram model for predicting the rates of OS, PFI, and DSS for glioma patients at 1, 3, and 5 years after surgery. The results showed that the calibration curves presented an optimal agreement between the prediction by nomogram and actual survival (Fig. 3E, Fig. S3B and S3D).

**Fig. 4:**
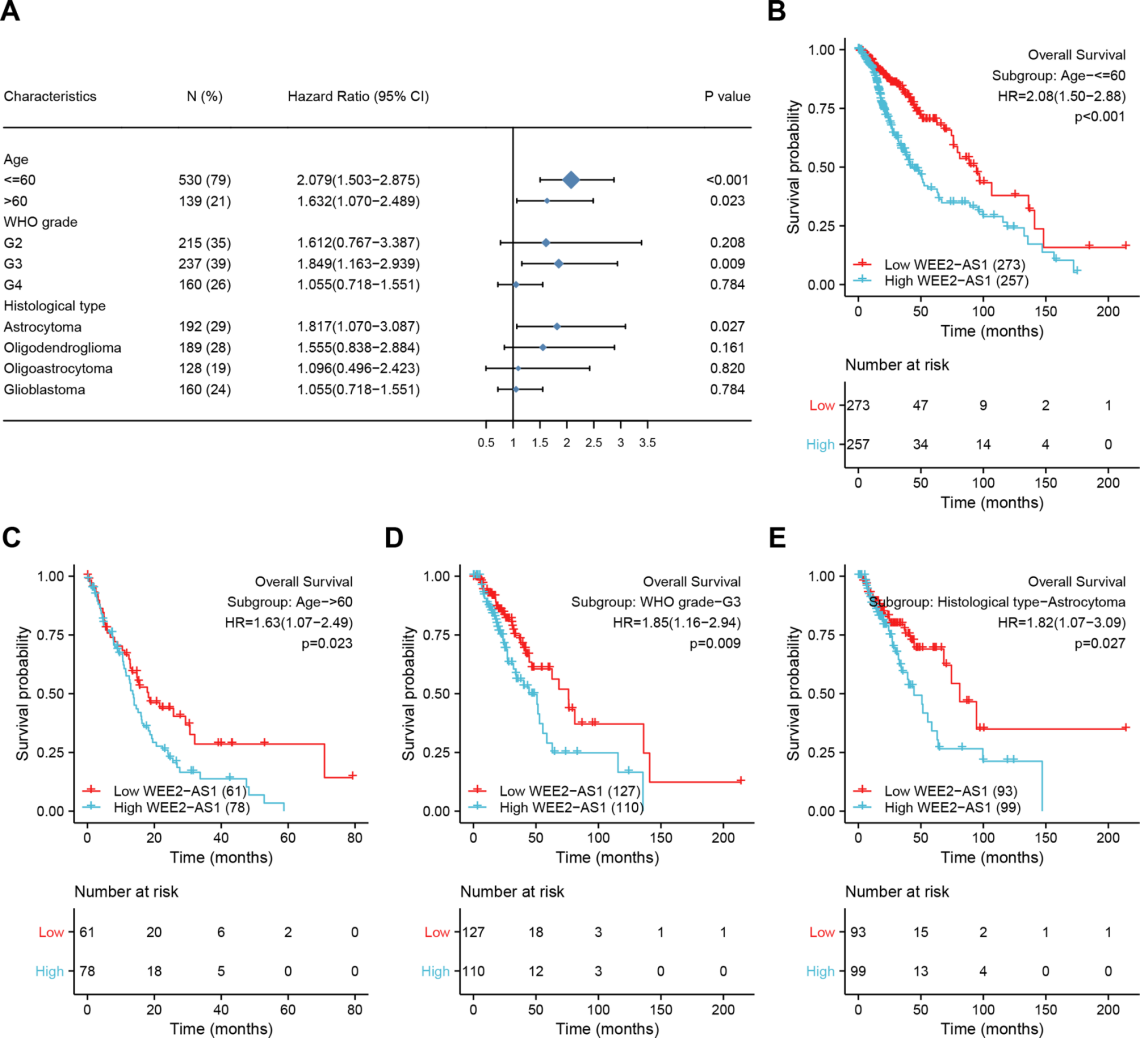
OS prognostic performance of WEE2-AS1 in clinicopathological subgroups (A) Forest plot of hazard ratios for the relationship between WEE2-AS1 and overal survival in TCGA cohort. (B)–(E) Kaplan–Meier plots of OS between expression of WEE2-AS1 and subgroups of patients with glioma in TCGA cohort. A high level of WEE2-AS1 is associated with poor survival outcomes in the age (B) and (C), G3 (D), and astrocytoma (E) subgroups

To further validate the role of WEE2-AS1 expression in the prognosis of patients with glioma, we performed subgroup survival analyses of OS, PFI, and DSS by forest plot and Kaplan–Meier plots. The prognostic value of OS in each subgroup of patients with glioma was statistically significant in age ≤ 60 (HR = 2.079, 95% CI = 1.503–2.875, p < 0.001), age > 60 (HR = 1.632, 95% CI = 1.070–2.489, p = 0.023), G3 (HR = 1.849, 95% CI = 1.163–2.939, p = 0.009), astrocytoma (HR = 1.817, 95% CI = 1.070–3.087, p = 0.027), IDH-wildtype (HR = 1.40, 95% CI = 1.01–1.95, p = 0.045) and 1p/19q non-codel (HR = 1.58, 95% CI = 1.20–2.08, p = 0.001) subgroups (Fig. 4 and Fig. S4). While, there is no difference in OS in G2, G4, oligodendroglioma, oligoastrocytoma, glioblastoma, IDH-mutant, and 1p/19q co-codel in the subgroups of patients with glioma (p > 0.05) (Fig. S4). The results showed the high expression levels of WEE2-AS1 were associated with OS outcomes in age, WHO G3 grade, astrocytoma, IDH-wildtype, and 1p/19q non-codel type with glioma. These results also apply to PFI and DSS (Fig. S5 and Fig. S6).

Overall, these results confirmed that the expression level of WEE2-AS1 was significantly related to the prognosis of OS, PFI, and DSS in patients with glioma.

### Identification of DEGs and functional enrichment analysis

**Fig. 5:**
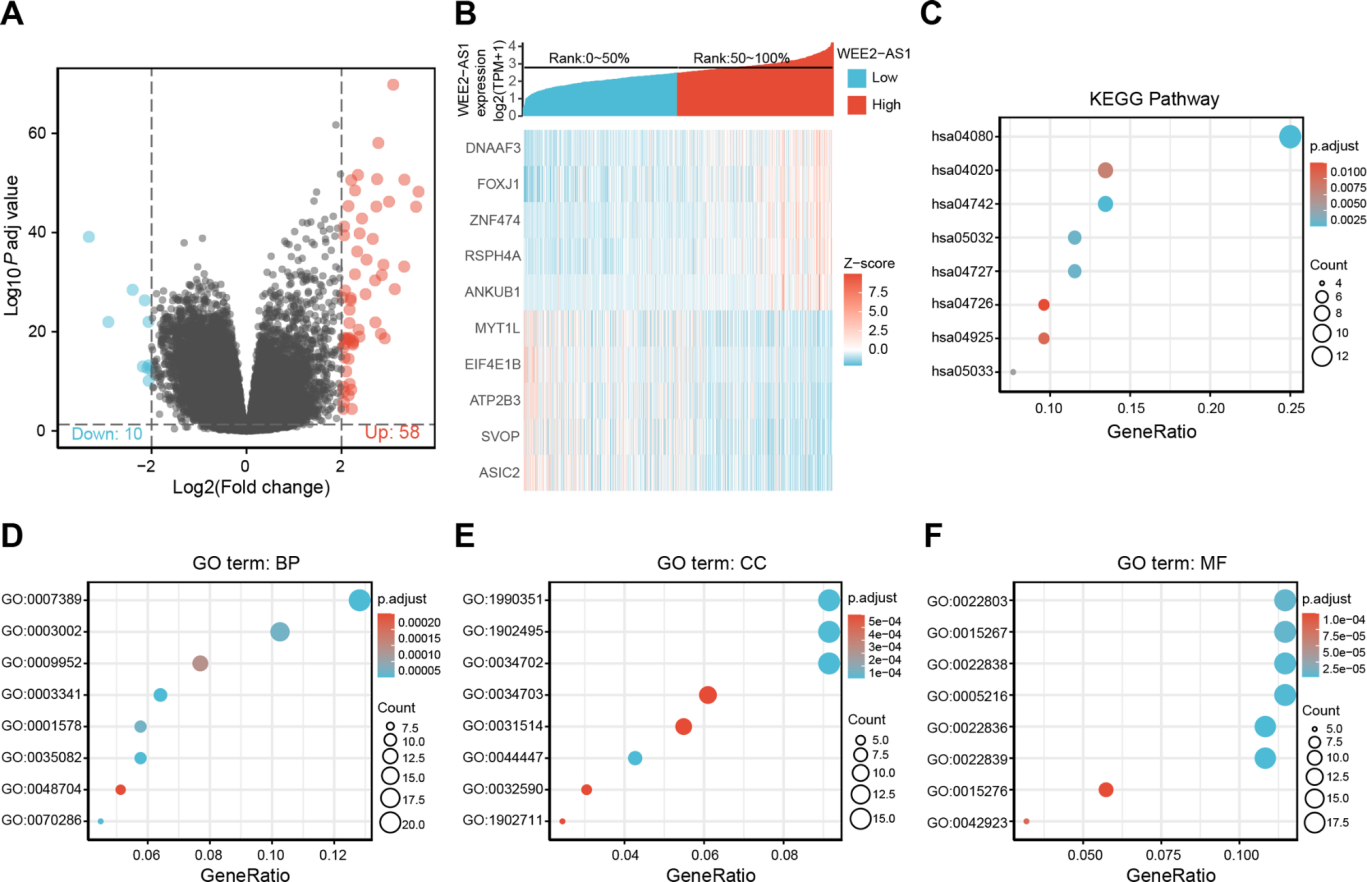
Identification of differentially expressed genes (DEGs) and functional enrichment analysis of WEE2-AS1 in glioma (A) Volcano plot of differentially expressed RNAs. Green and red represent upregulated and downregulated genes, respectively. The 68 differential molecules had log2FC < -2 and adjusted p < 0.05. (B) Heat map of the top 10 co-expressed differential genes in the TCGA dataset. (C) Kyoto Encyclopedia of Genes and Genomes (KEGG) enrichment analysis results. (D)–(F) Gene Ontology (GO) enrichment analysis results in the biological process (BP), Cellular Component (CC), and Molecular Function (MF) categories. The X-axis represents the proportion of DEGs and the Y-axis represents different categories. The different colors indicate different properties and the different sizes represent the numbers of DEGs.

From the above results, we learned that WEE2-AS1 plays a central role in the progression and prognosis of glioma, but the specific molecular mechanism was still unclear. To reveal the mechanism of WEE2-AS1 in gliomas, we first divided HTSeq-counts data into high- and low- WEE2-AS1 expression groups based on the cutoff criteria. A total of 68 upregulated and downregulated genes were identified based on |log2FC| > 2 and adjusted p < 0.05 (Fig. 5A). DEGs in HTSeq-counts were further analyzed using the DESeq2 package. Relative expression values of the top 10 DEGs between the two cohorts are shown in Fig. 5B.

To analyze the functional implications of WEE2-AS1 in glioma, we performed KEGG and GO enrichment analyses. According to the KEGG analysis results, neuroactive ligand-receptor interaction, GABAergic synapse, calcium signaling pathway, aldosterone synthesis and secretion, and serotonergic synapse were remarkably enriched (Fig. 5C). The GO function analysis of DEGs was divided into three parts: BP, CC, and MF. The GO analysis results showed that co-expressed genes were primarily closely related to the biological process of extracellular matrix remodeling (Fig. 5D-F).

**Fig. 6:**
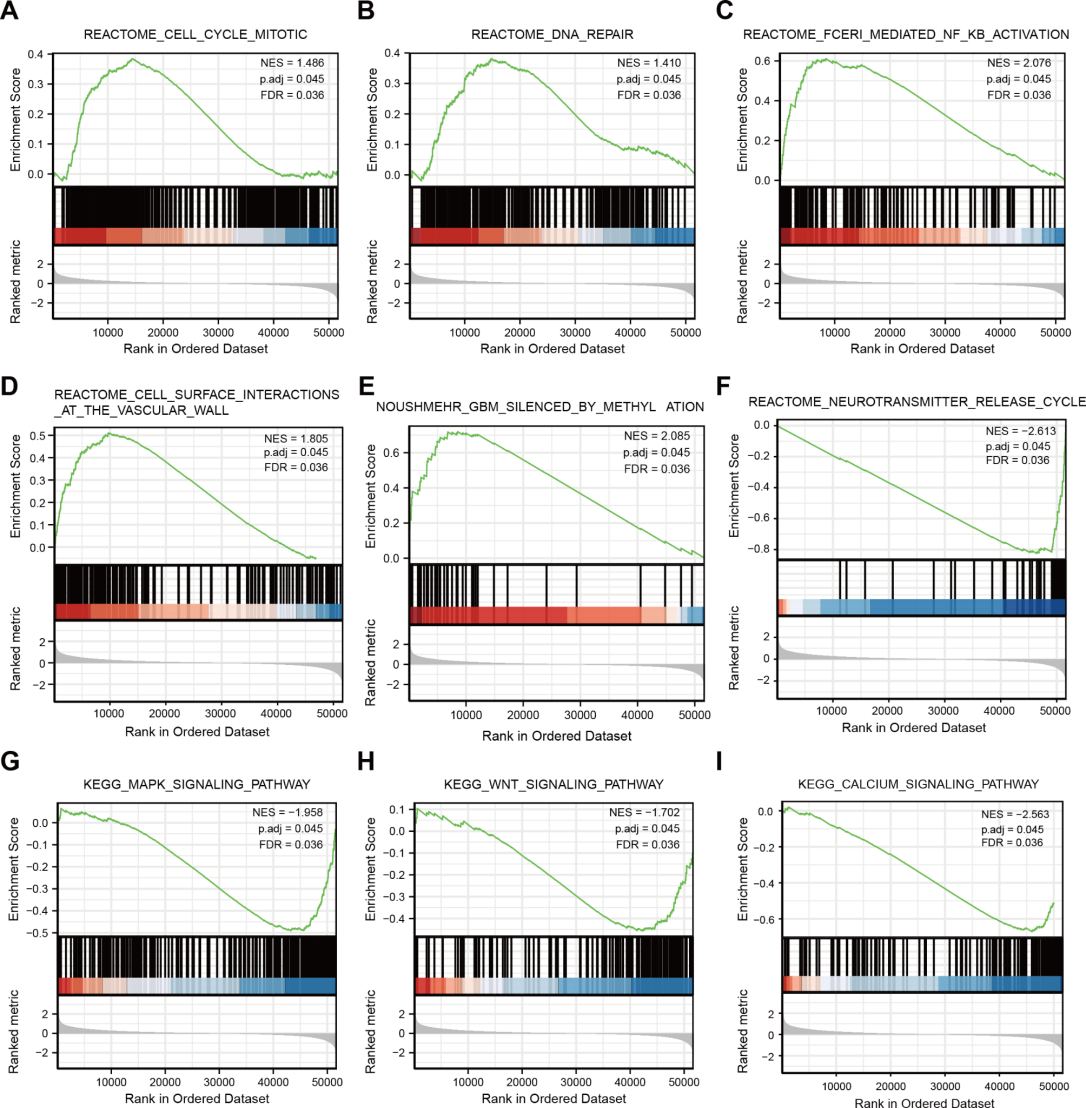
GSEA analysis results of the TCGA RNA-seq data (A)–(E) The cell cycle mitotic (A), DNA repair (B), Fc epsilon receptor I (FCERI)-mediated nuclear factor kappa light chain enhancer of activated B cells (NF-κB) activation (C), cell surface interactions at the vascular wall (D), and GBM silenced by methylation (E) pathways were differentially enriched the in WEE2-AS1 high-expression phenotype. (F)–(I) In the WEE2-AS1 low-expression phenotype, enriched pathways included the neurotransmitter release cycle (F), mitogen-activated protein kinase (MAPK) signaling (G), Wnt signaling pathways (H) and calcium signaling pathways (I)

To further elucidate the mechanism of WEE2-AS1 in gliomas, we used gene set enrichment analysis (GSEA) [26] to evaluate the potential signaling pathway used by WEE2-AS1 in gliomas. 670 glioma tissues data from TCGA (GBM-LGG) database were analyzed with the clusterProfiler package [22], in which C2.all.v7.0.symbols.gmt [Immunologic signatures] was selected from MSigDB Collections as the reference gene collection; an FDR < 0.25 and an adjusted p < 0.05 were considered significantly enriched. These results revealed that the cell cycle mitotic, DNA repair, Fc epsilon RI (FCERI)-mediated NFkB activation, cell surface interactions at the vascular wall, and GBM silenced by methylation were differentially enriched in the WEE2-AS1 high-expression phenotype (Fig. 6A–E). In the WEE2-AS1 low-expression phenotype, enriched pathways included the neurotransmitter release cycle, mitogen-activated protein kinase (MAPK), the wingless-related integration site (Wnt), and calcium signaling pathways (Fig. 6F-I). In addition, we also assessed the underlying mechanisms of the negative prognostic role of WEE2-AS1 in gliomas by GSEA according to the different grades of gliomas. These signaling pathways of cell surface interactions at the vascular wall, neurotransmitter release cycle, MAPK and calcium also apply to low-grade glioma (Fig. S7) and GBM (Fig. S8).

### Correlation between WEE2-AS1 and the immune infiltration of glioma

**Fig. 7:**
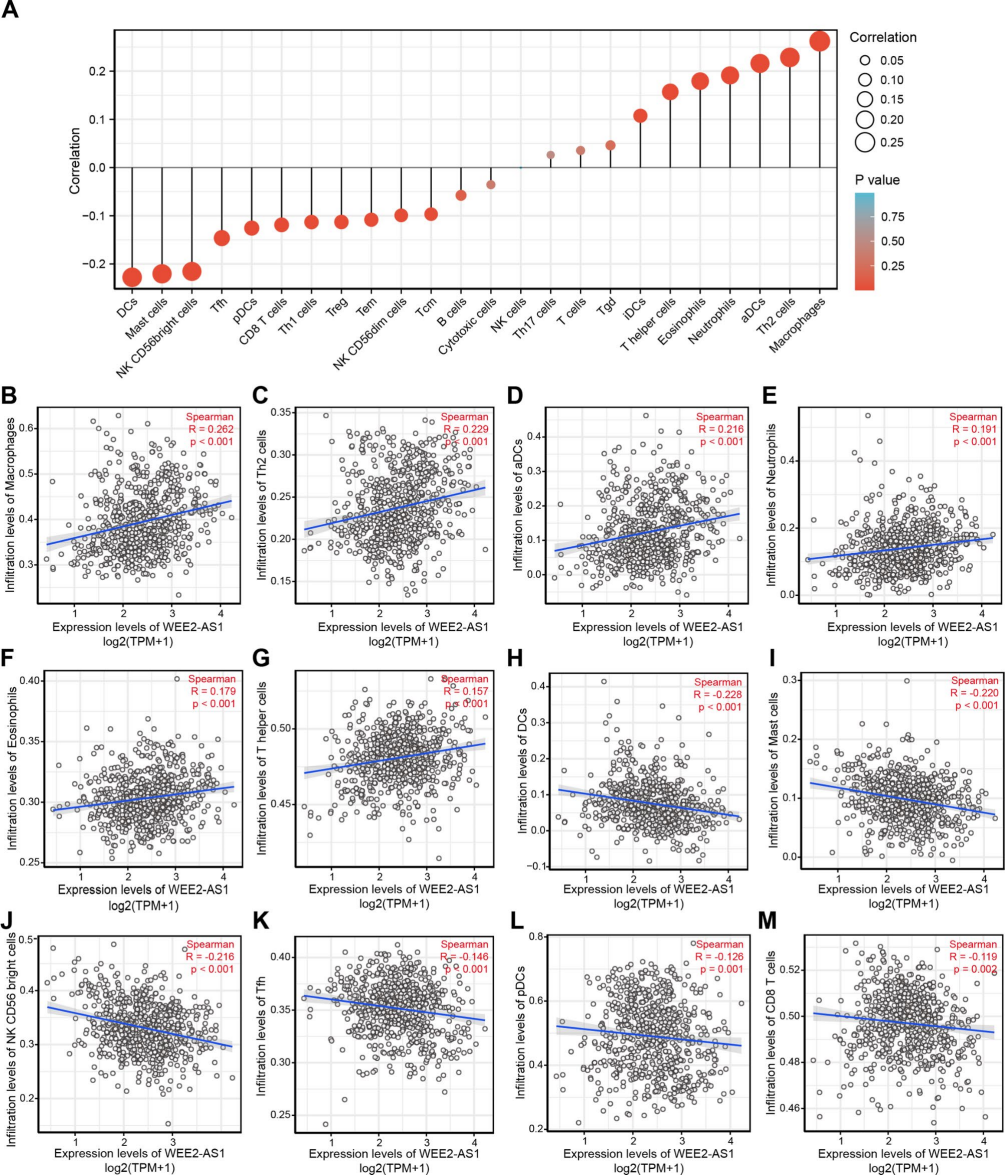
Correlation between WEE2-AS1 and the immune infiltration of glioma (A) Correlation between the relative abundance of 24 types of immune cells and WEE2-AS1 expression level. The size of dots shows the absolute value of Spearman’s rank correlation. (B)–(M) Correlation between the expression level expressed as transcripts per kilobase million (TPM) of WEE2-AS1 and the relative enrichment score of dendritic cells (DCs) (B), mast cells (C), natural killer CD56 bright cells (D), T follicular helper (Tfh) cells (E), plasmacytoid dendritic cells (pDCs) (F), CD8 T cells (G), T helper cells (H), eosinophils (I), neutrophils (J), activated dendritic cells (aDCs) (K), Th2 cells (L), and macrophages (M)

Immune infiltration is a vital factor in the progression of glioma that significantly affects the survival rate of patients with glioma [1, 32]. We used the ssGSEA method to analyze the relationship between the expression of WEE2-AS1 and the infiltration of 24 types of immune cells in glioma [27]. We observed that WEE2-AS1 expression was significantly positively correlated with macrophages (R = 0.262, p < 0.001), helper T2 (Th2) cells (R = 0.229, p < 0.001), activated dendritic cells (aDCs; R = 0.216, p < 0.001), neutrophils (R = 0.191, p < 0.001), eosinophils (R = 0.179, p < 0.001), and T helper (Th) cells (R = 0.157, p < 0.001). However, DCs (R = − 0.228, p < 0.001), mast cells (R = − 0.220, p < 0.001), natural killer CD56 bright cells (R = − 0.216, p < 0.001), T follicular helper (Tfh) cells (R = − 0.146, p < 0.001), plasmacytoid dendritic cells (pDCs; R = − 0.126, p = 0.001), and CD8 T cells (R = − 0.119, p = 0.002) were negatively associated with WEE2-AS1 expression (Fig. 7). In summary, these results indicated that WEE2-AS1 influences the infiltration of immune cells into the glioma microenvironment.

## Discussion

In this study, we explored the correlation between WEE2-AS1 and glioma using TCGA and GTEx databases. We found that WEE2-AS1 was overexpressed in glioma and the elevated WEE2-AS1 was related to advanced clinicopathological features. We observed that the expression of WEE2-AS1 in patients with glioma may be an independent risk factor for reduced progression-free survival and overall survival. Moreover, WEE2-AS1 could influence the cell cycle and infiltration of immune cells into the glioma microenvironment. These results hint that WEE2-AS1 is a promising biomarker for the diagnosis and prognosis of patients with glioma and provides a new hope for improving the survival and prognosis of patients with glioma.

The survival rate of patients with glioma is poor because of the high morbidity, recurrence rate, and mortality of glioma [2]. In recent years, with the in-depth understanding of the pathogenesis of glioma, the survival rate of patients with glioma has improved to a certain extent, but there is still much room for improvement [6]. Therefore, understanding the specific mechanisms of the occurrence of glioma from the molecular biology level, and then finding effective biomarkers for early diagnosis and treatment of glioma, are of great significance for improving the clinical prognosis of patients. Taken together, our findings suggested WEE2-AS1 may be involved in the malignant progression of glioma and have a high diagnostic value for glioma. WEE2-AS1 expression value could also be a stable factor for predicting the prognosis of patients with glioma.

Long non-coding RNAs (lncRNAs) are non-coding RNAs with more than 200 nucleotides [33]. Recent studies have identified a wide range of lncRNAs involved in the occurrence and progression of glioma and these lncRNAs are becoming new targets for the diagnosis and treatment of glioma [34–36]. WEE2-AS1, as a lncRNA, has been previously reported to play a critical role in various cancer types, promote cancer cell proliferation and invasion, and may act as an oncogenic gene [15–17]. WEE2-AS1 contributes to the proliferation, migration, and invasion of hepatocellular carcinoma cells via regulating the fermitin family member 3 pathway and activating the PI3K/AKT/GSK3b signaling pathway [15]. Lin et al. found that depletion of WEE2-AS1 impaired tumor growth and attenuated GBM cell proliferation and invasion [17]. However, the utility of WEE2-AS1 as a potential glioma diagnostic and prognostic marker has not been fully clarified.

To address this problem in the current study, bioinformatic analyses were performed to investigate the function and value of WEE2-AS1 in glioma. We clarified the crucial role of WEE2-AS1 in the prognosis of glioma and verified it as a reliable biomarker for the diagnosis and prognosis of glioma. First, the data of TCGA and GTEx databases showed that the expression of WEE2-AS1 was upregulated in glioma, and the results of RT-qPCR also demonstrated that the expression of WEE2-AS1 was upregulated in both glioma cells and tissue. We further investigated the relationship between the clinicopathological features of patients with glioma and found that the expression of WEE2-AS1 was positively correlated with WHO grade, histological type, IDH status, 1p/19q codeletion status, and EGFR status of patients with glioma. The results of ROC curve, Kaplan–Meier survival analysis, univariate and multivariate Cox regression analyses confirmed that WEE2-AS1 could be used as an independent factor to predict poor prognosis in patients with glioma. In addition, the results of subgroup survival analyses showed that the prognostic value of OS was statistically significant in the astrocytoma subgroup of glioma, suggesting that WEE2-AS1 could be an important prognostic biomarker for the patients with glioma, especially astrocytoma. The patients of astrocytoma subgroup glioma may be more likely to benefit from the detection of WEE2-AS1.

For a more accurate prognostic prediction, nomograms that have been shown to be relatively accurate in predicting prognosis in some cancers were established [16, 29]. The C-index was used to evaluate the pretesting capability of the module, and our results showed that the C-indexes of the OS, PFI, and DSS were 0.858, 0.745, and 0.866, respectively. In addition, the calibration curves presented an optimal agreement between the prediction by nomogram and actual survival, which demonstrated that the nomogram performed well in predicting the survival in patients with glioma. Hence, our findings suggested that WEE2-AS1 can be used as a potential diagnostic and prognostic factor in patients with glioma. However, the mechanism that WEE2-AS1 led to an unfavorable prognosis of glioma needs to be further elucidated.

GSEA has been widely used to reveal the potential molecular mechanisms of specific genes in pathological processes of disease with high reliability [29]. Compared with traditional analysis methods, GSEA has the advantage of using a large sample size, which avoids the inherent deviation of experimental results caused by artificially setting thresholds [10]. To explore how WEE2-AS1 is involved in the malignant progress of glioma, we carried out GSEA analysis based on TCGA RNA-Seq data and found that WEE2-AS1 was significantly enriched in the MAPK and Wnt signaling pathways, and cell cycle mitotic and DNA repair. Accumulating evidence suggests that these signaling pathways play a crucial role in the occurrence and progression of glioma. For instance, the MAPK signaling pathway plays a vital role in the proliferation, migration, and invasion of glioma cells and affects the prognosis of patients with glioma [37]. Similarly, the abnormal activation of the Wnt signaling pathway promotes the cell autophagy and apoptosis of glioma and leads to poor survival in patients with glioma [38, 39]. Moreover, cell cycle mitotic and DNA repair also play an important role in cell differentiation, survival, proliferation, stem cell renewal, and cell fate determination during development and morphogenesis [40, 41]. Taken together, these results suggest that WEE2-AS1 affects the prognosis of glioma through the tumor-associated signaling pathways described above.

It is known that the occurrence and development of glioma are associated with immune cell infiltration and activation, which have been shown to contribute to tumor progression and response to immunotherapy [10, 42]. Numerous studies have reported significant progress in the clinical application of immunotherapy for glioma, which has become another effective treatment method following surgery, radiotherapy, and chemotherapy [32, 43]. However, no studies have reported the association of WEE2-AS1 with immune infiltration in glioma. In our study, we found that the expression of WEE2-AS1 was strongly correlated with macrophages and DCs in glioma. A previous study showed that colony-stimulating factor 1 receptor inhibition alters macrophage polarization and blocks glioma progression [44]. Wang et al. showed that nuclear factor-erythroid factor 2-related factor 2 facilitates the escape of glioma cells from the immune system through suppressing DC function [45]. These results suggest that WEE2-AS1 may exert its oncogene effect by affecting the function of macrophages and DCs. Hence, our results confirmed that WEE2-AS1 is involved in the tumor immune microenvironment primarily through regulating the functions of macrophages and DCs and that WEE2-AS1 may affect the survival rate of patients with glioma by affecting the tumor immune microenvironment. These results further illustrated the complexity of the mechanism of WEE2-AS1 in glioma. Moreover, they also clarified the potential biological pathway regulation mechanism that WEE2-AS1 may participate in the regulation of glioma.

However, our study has some inevitable limitations. The sample data were obtained from the TCGA and GTEx databases and did not provide specific information on the extent of surgical resection, which is a key factor for the survival of patients with glioma. In addition, there are some known issues with TCGA data, such as bias in glioma types, which may contribute to the reason why WEE2-AS1 is not a universal prognostic marker for all glioma subtypes. However, WEE2-AS1 may have important value for the clinical prognosis of patients with glioma, especially astrocytoma. Moreover, we lacked sufficient clinical data to validate the predictive value of WEE2-AS1 in response to glioma immunotherapy. Therefore, further analysis with more detailed clinical information should be conducted. Nevertheless, it is important to note that we have analyzed the correlation between WEE2-AS1 and glioma at multiple levels and multiple databases to ensure the comprehensiveness and reliability of our research. Our results identified WEE2-AS1 as a promising biomarker for the diagnosis and prognosis of patients with glioma.

## Conclusion

In conclusion, this study revealed that the expression of WEE2-AS1 is positively correlated with the clinical features of glioma and the mortality rate in patients with glioma. WEE2-AS1 is a promising prognostic biomarker because its upregulation is predictive of a poor prognosis for patients with glioma. Moreover, WEE2-AS1 potentially promotes the process of malignant growth in glioma through interactions with tumor-related pathways, such as the cell cycle and regulation of the immune microenvironment. Therefore, WEE2-AS1 plays an important role as a novel biomarker in the prognosis of glioma. An increased understanding of its effects on the regulation of cell growth may lead to the development of clinical applications that improve the prognostic status of patients with glioma.

## Electronic supplementary material

Below is the link to the electronic supplementary material.

Supplementary Material 1

Supplementary Material 2

Supplementary Material 3

Supplementary Material 4

Supplementary Material 5

Supplementary Material 6

Supplementary Material 7

Supplementary Material 8

Supplementary Material 9

Supplementary Material 10

## Data Availability

The datasets generated during the current study are available in the TCGA repository (https://portal.gdc.cancer.gov/), UCSC Xena (http://xenabrowser.net/datapages/) and CGGA (http://www.cgga.org.cn/) database. The datasets were processed using the rms package (version 5.1-3; http://cran.rproject.org/w-eb/packages/rms/index.html). This study was entirely following the publication guidelines provided by TCGA, GTEx and CGGA. All the data were available from the corresponding authors for reasonable request.

## Abbreviations

TCGA: The Cancer Genome Atlas
GSEA: Gene set enrichment analysis
CGGA: Chinese glioma genome atlas
DEGs: Differentially Expressed Genes
MGMT: O-6-methylguanine-DNA methyltransferase
EGFR: Epidermal growth factor receptor
IDH: Isocitrate dehydrogenase
OS: Overall Survival
DSS: Disease Specific Survival
GO: Gene Ontology
KEGG: Kyoto Encyclopedia of Genes and Genomes
GSEA: Gene set enrichment analysis
ROC: Receiver operating characteristic
RT-QPCR: Real-time quantitative polymerase chain reaction
