# Supplementary figures and images for: LncRNA WEE2-AS1 is a diagnostic biomarker that predicts poor prognoses in patients with glioma
Source: BMC Cancer. 2023 Feb 6;23:120. doi: 10.1186/s12885-023-10594-y (PMC9901081; doi:10.1186/s12885-023-10594-y)

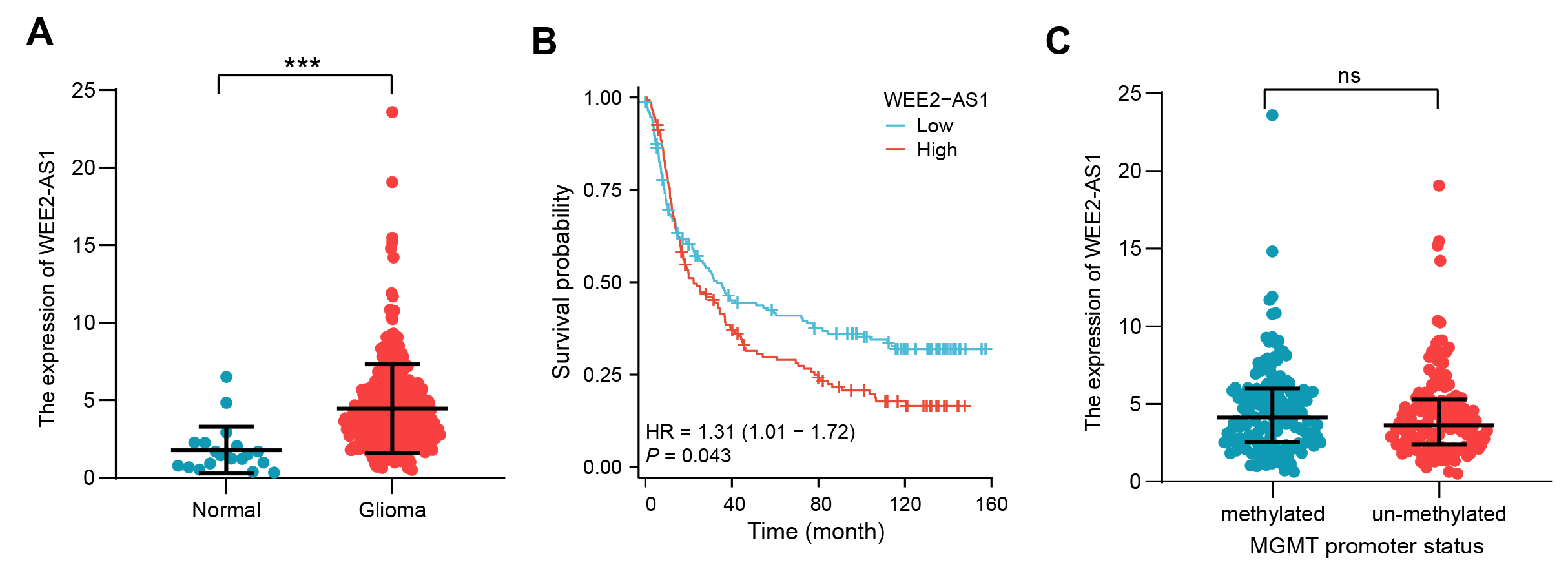

Supplement: Supplementary file 1 — Supplementary Material 1 [file 12885_2023_10594_MOESM1_ESM.tif]

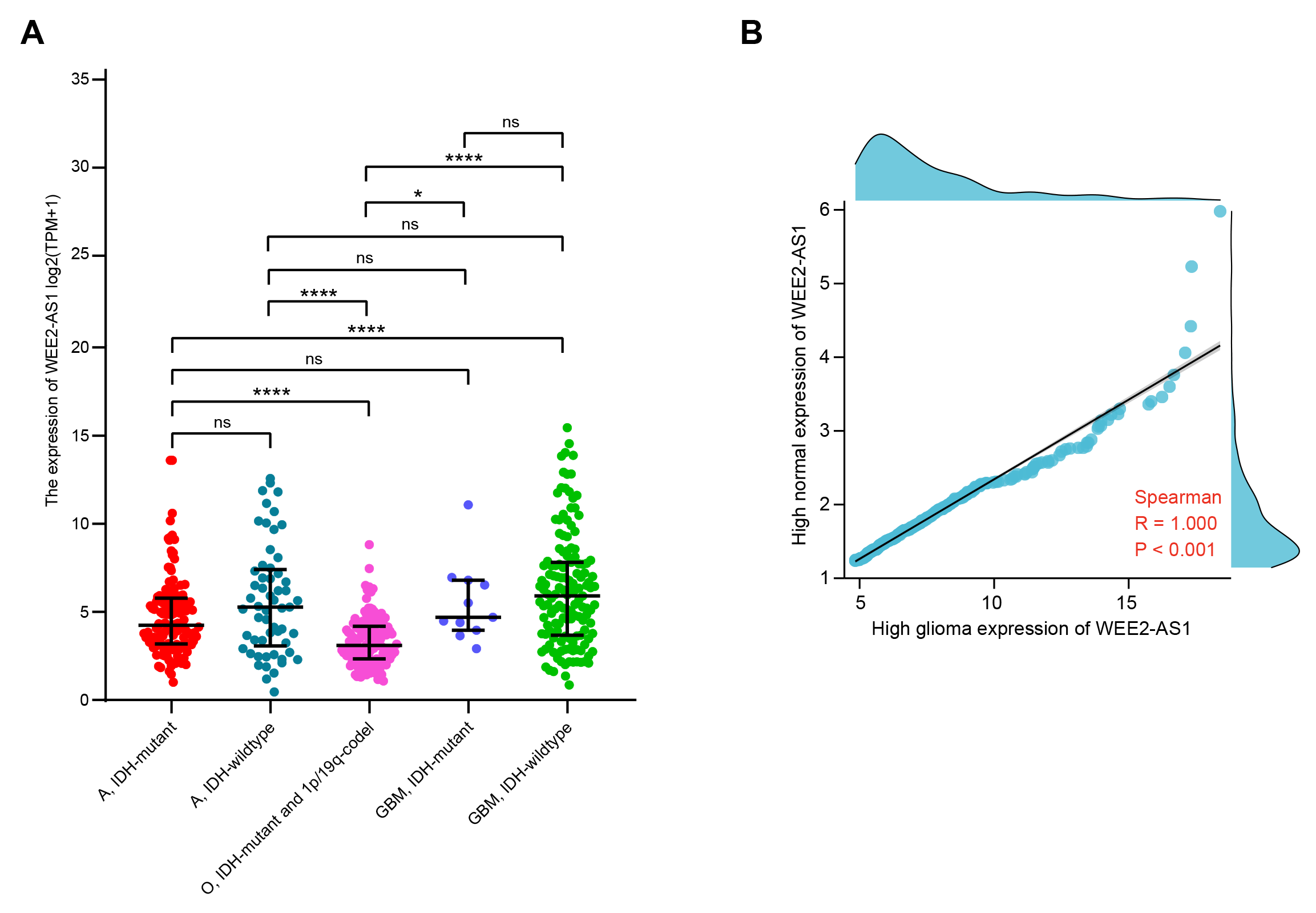

Supplement: Supplementary file 2 — Supplementary Material 2 [file 12885_2023_10594_MOESM2_ESM.tif]

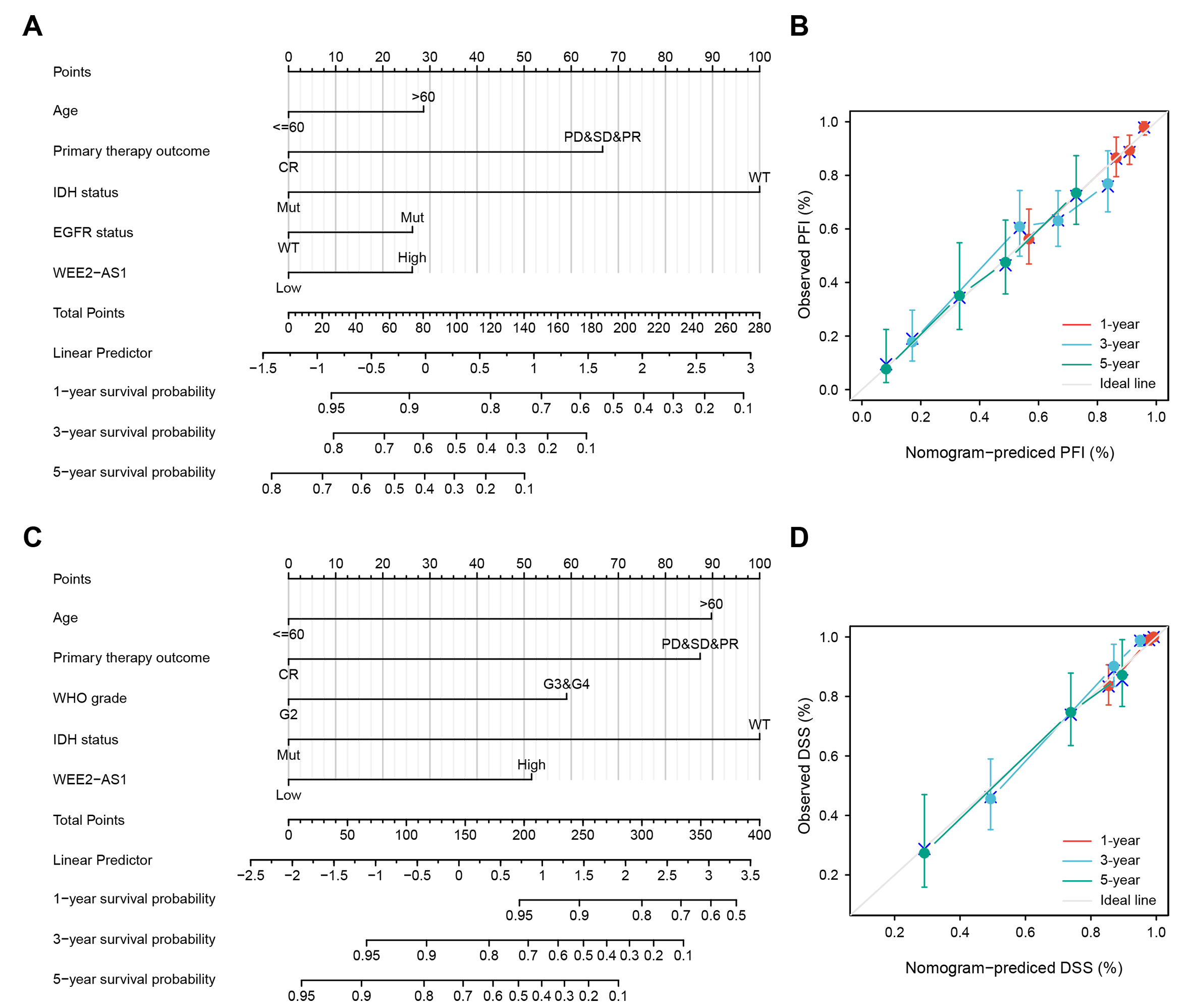

Supplement: Supplementary file 3 — Supplementary Material 3 [file 12885_2023_10594_MOESM3_ESM.tif]

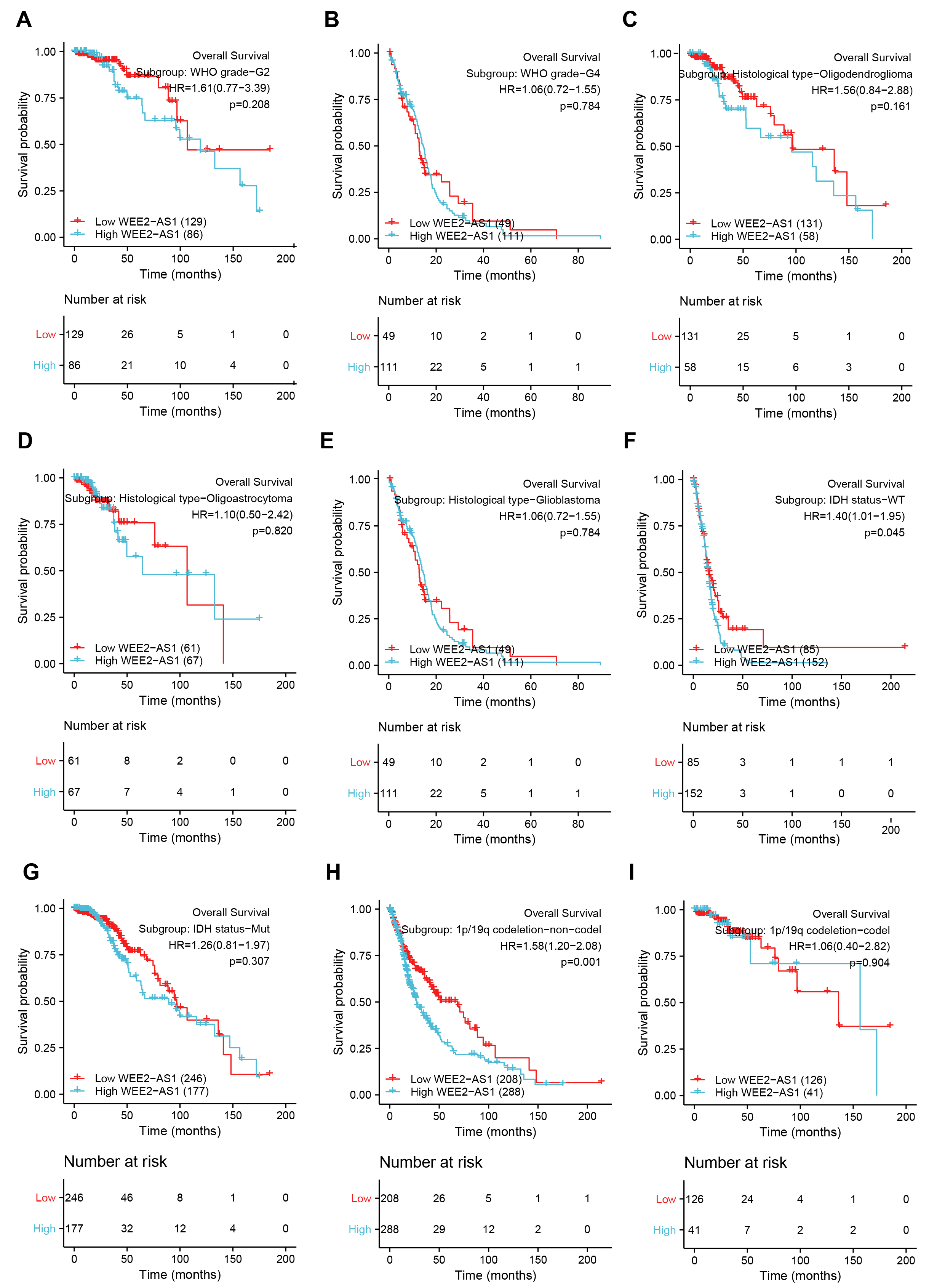

Supplement: Supplementary file 4 — Supplementary Material 4 [file 12885_2023_10594_MOESM4_ESM.tif]

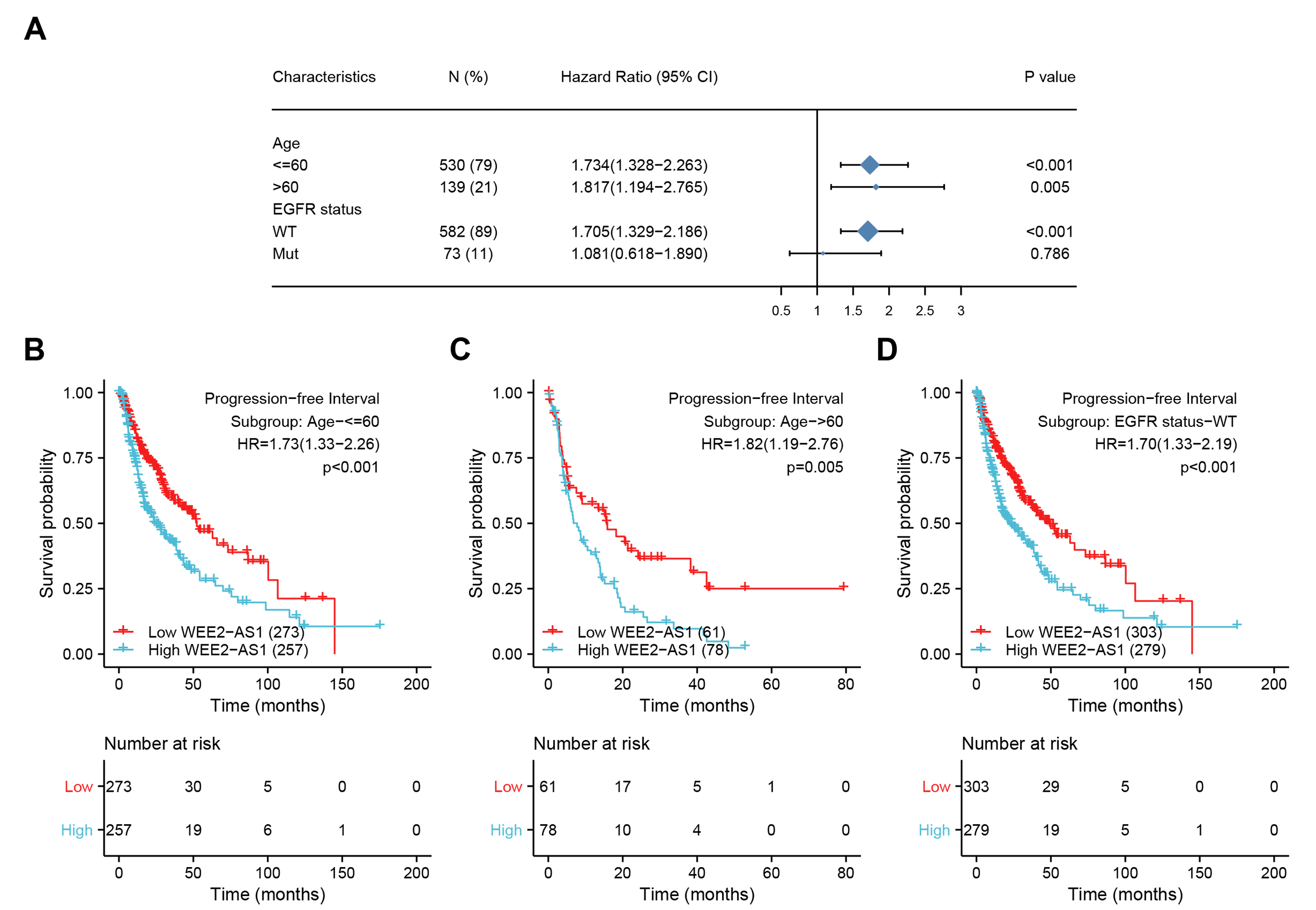

Supplement: Supplementary file 5 — Supplementary Material 5 [file 12885_2023_10594_MOESM5_ESM.tif]

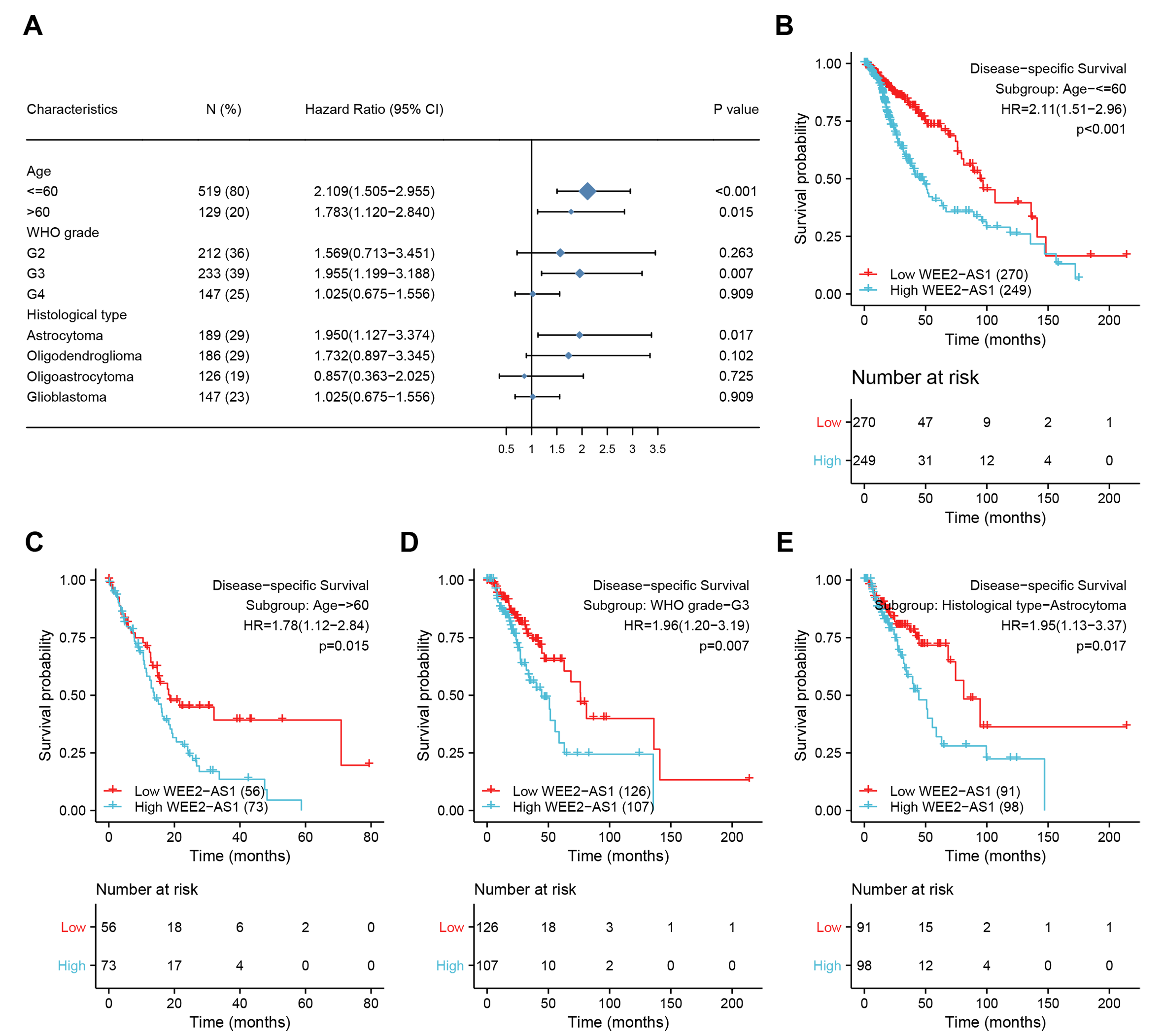

Supplement: Supplementary file 6 — Supplementary Material 6 [file 12885_2023_10594_MOESM6_ESM.tif]

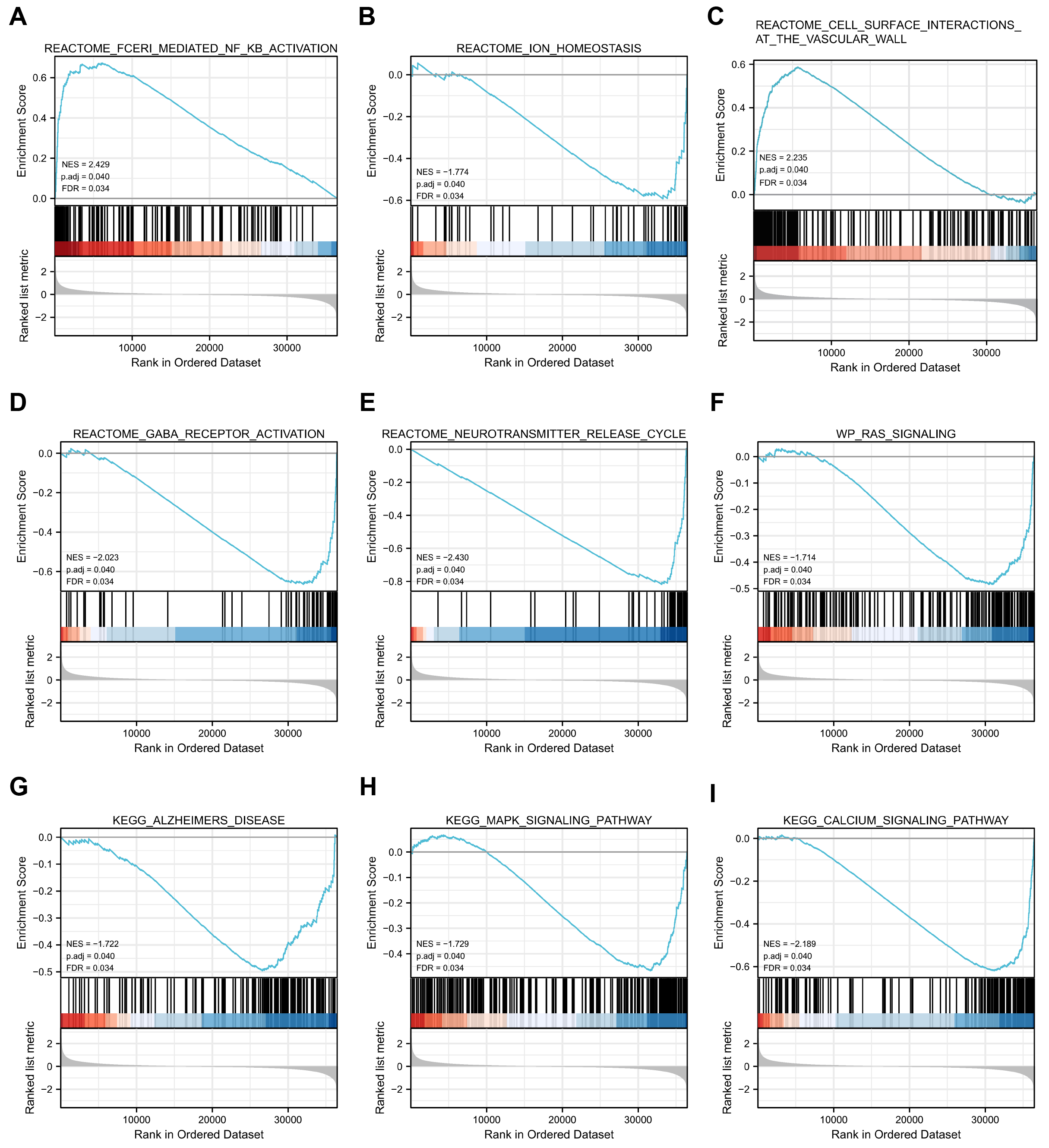

Supplement: Supplementary file 7 — Supplementary Material 7 [file 12885_2023_10594_MOESM7_ESM.tif]

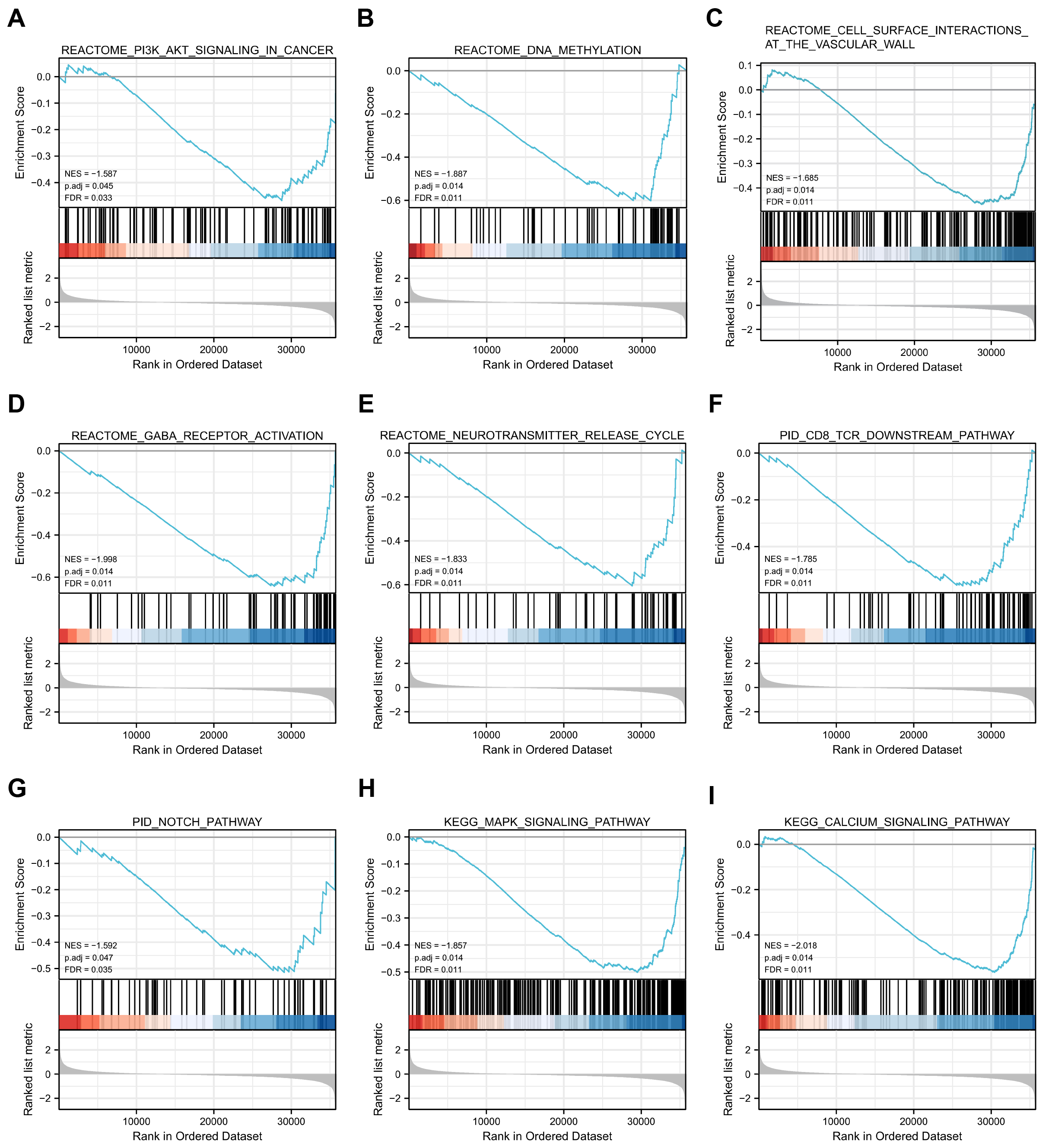

Supplement: Supplementary file 8 — Supplementary Material 8 [file 12885_2023_10594_MOESM8_ESM.tif]
