# Supplementary material for: LncRNA WEE2-AS1 is a diagnostic biomarker that predicts poor prognoses in patients with glioma
Source: BMC Cancer. 2023 Feb 6;23:120. doi: 10.1186/s12885-023-10594-y (PMC9901081; doi:10.1186/s12885-023-10594-y)
Supplement: Supplementary file 9 — Supplementary Material 9 [file 12885_2023_10594_MOESM9_ESM.docx]

Supplementary Figure 1 The expression of WEE2-AS1 and overall survival of patients with glioma from Chinese glioma genome atlas (CGGA).

(A) The expression levels of WEE2-AS1 in 325 glioma tissues and 20 normal brain tissues from RNA-Seq data in CGGA databases (unpaired t test, p < 0.001). (B) Kaplan–Meier survival analysis showing that effect of WEE2-AS1 expression level on overall survival (OS) in patients with glioma in CGGA databases. (C) The relationship between the WEE2-AS1 expression and MGMT promoter status (unpaired t test, p > 0.05). Data are represented as median ± upper and lower interquartile range. ns, no significance; ***p < 0.001.

Supplementary Figure 2 Correlations between WEE2-AS1 and pathological characteristics of glioma

(A) The correlation between the expression of WEE2-AS1 and pathological characteristics of patients with glioma from TCGA according to the 2016 WHO classification system (oneway ANOVA followed by Tukey's multiple-comparisons test). (B) The correlation between 342 normal samples of high WEE2-AS1 expression and 342 tumor samples of high WEE2-AS1 expression using the available sets of paired normal + tumor glioma samples from TCGA and GTEx (spearman, r = 1.000, p < 0.001. Data are represented as median ± upper and lower interquartile range. ns, no significance; *p < 0.05, ****p < 0.0001.

Supplementary Figure 3 The relationship between the level of WEE2-AS1 and the progression-free interval (PFI) and disease-specific survival (DSS) of patients with glioma

(A) Nomogram for predicting the probability of 1-, 3-, and 5-year PFI for patients with glioma. (B) Calibration plot of the nomogram for predicting the probability of PFI at 1, 3, and 5 years. The abscissa is the probability of the prognosis predicted by the model (0–1 indicates that the probability of the event occurring is 0–100%) and the ordinate is the actual observed prognosis. The colored line is the fitted line indicating the predicted value (the horizontal axis) corresponding to the actual value (the vertical axis). The gray diagonal is the ideal case.

Supplementary Figure 4 OS prognostic performance of WEE2-AS1 in clinicopathological subgroups

(A)–(I) Kaplan–Meier plots of OS between expression of WEE2-AS1 and subgroups of patients with glioma in TCGA cohort. A high level of WEE2-AS1 is associated with poor survival outcomes in the IDH-wildtype (F) and 1p/19q non-codel (H) subgroups. There is no difference in OS in G2 (A), G4 (B), oligodendroglioma (C), oligoastrocytoma (D), glioblastoma (E), IDH-mutant (G), and 1p/19q co-codel (I) in the subgroups of patients with glioma.

Supplementary Figure 5 PFI prognostic performance of WEE2-AS1 in clinicopathological subgroups

(A) Forest plot of hazard ratios for the relationship between WEE2-AS1 and PFI in The Cancer Genome Atlas (TCGA) cohort. (B)–(D) Kaplan–Meier plots of PFI between expression of WEE2-AS1 and subgroups of patients with glioma in TCGA cohort. High levels of WEE2-AS1 are associated with poor survival outcomes in the age (B) and (C) and epidermal growth factor receptor (EGFR) status (D) subgroups.

Supplementary Figure 6 DSS prognostic performance of WEE2-AS1 in clinicopathological subgroups

(A) Forest plot of hazard ratios for the relationship between WEE2-AS1 and DSS in TCGA cohorts. (B)–(D) Kaplan–Meier plots of DSS between expression of WEE2-AS1 and subgroups of patients with glioma in TCGA cohorts. High level of WEE2-AS1 is associated with poor survival outcomes in the age (B) and (C), G3 (D), and astrocytoma (E) subgroups.

Supplementary Figure 7 GSEA analysis results of LGG patients from the TCGA data

(A) and (C) The Fc epsilon receptor I (FCERI)-mediated nuclear factor kappa light chain enhancer of activated B cells (NF-κB) activation (A) and cell surface interactions at the vascular wall (C) pathways were differentially enriched the in WEE2-AS1 high-expression phenotype. (B) and (D)-(I) In the WEE2-AS1 low-expression phenotype, enriched pathways included the ion homeostasis (B), Gaba receptor activation (D), neurotransmitter release cycle (E), Ras (F), alzheimers disease (G), mitogen-activated protein kinase (MAPK) signaling (H), and calcium signaling pathways (I).

Supplementary Figure 8 GSEA analysis results of HGG patients from the TCGA data

(A)-(I) In the WEE2-AS1 low-expression phenotype, enriched pathways included the PI3K/AKT signaling in cancer (A), DNA methylation (B), cell surface interactions at the vascular wall (C), Gaba receptor activation (D), neurotransmitter release cycle (E), CD8 TCR downstream (F), Notch (G), mitogen-activated protein kinase (MAPK) signaling (H), and calcium signaling pathways (I).
