# Supplementary material for: LncRNA WEE2-AS1 is a diagnostic biomarker that predicts poor prognoses in patients with glioma
Source: BMC Cancer. 2023 Feb 6;23:120. doi: 10.1186/s12885-023-10594-y (PMC9901081; doi:10.1186/s12885-023-10594-y)
Supplement: Supplementary file 10 — Supplementary Material 10 [file 12885_2023_10594_MOESM10_ESM.docx]

Supplementary Table S1 The clinicopathological characteristics of 10 patients with glioma in our dataset.

| **Characteristics** | **Number** |
| --- | --- |
| **Age** | **10** |
| <=60 | 6 |
| >60 | 4 |
| **Gender** | **10** |
| Male | 5 |
| Female | 5 |
| **WHO grade**  G2  G3  G4  **Histology** | **10** |
|  | 3 |
|  | 3 |
|  | 4 |
|  | **10** |
| A, IDH-mutant | 1 |
| A, IDH-wildtype | 1 |
| AA, IDH-mutant | 1 |
| AA, IDH-wildtype | 1 |
| O, IDH-mutant and 1p/19q codel | 1 |
| AO, IDH-mutant and 1p/19q codel | 1 |
| GBM, IDH-mutant | 1 |
| GBM, IDH-wildtype | 3 |
| **IDH status** | **10** |
| Mutant | 5 |
| Wild type | 5 |
| **1p/19q codeletion** | **8** |
| Codel | 2 |
| Non-codel | 6 |
| **MGMT promoter status** | **7** |
| Methylated | 2 |
| Unmethylated | 5 |

A, astrocytoma; AA, anaplastic astrocytoma; O, oligodendroglioma; AO, anaplastic oligodendroglioma; GBM, glioblastoma.

Supplementary Table S2 The clinicopathological characteristics of 325 patients with glioma in CGGA dataset.

| **Characters** | **Level** | **Number** |
| --- | --- | --- |
| N |  | 325 |
| Age | <=60 | 301 |
|  | >60 | 24 |
| Gender | Female | 122 |
|  | Male | 203 |
| WHO grade | G2 | 103 |
|  | G3 | 79 |
|  | G4 | 139 |
| Histological type | Astrocytoma  Anaplastic astrocytoma | 56  62 |
|  | Oligodendroglioma  Anaplastic oligodendroglioma  Glioblastoma | 52  12  139 |
| IDH status | Mut | 175 |
|  | WT | 149 |
| 1p/19q codeletion | Codel | 67 |
|  | Non-codel | 250 |
| MGMT promoter status | methylated | 157 |
|  | un-methylated | 149 |

Supplementary Table S3 The expression levels of WEE2-AS1 in 33 tumor tissues from GTEx and TCGA databases.

| **Tumors** | **Groups** | **Number** | **Median** | **IQR** | ***P***^*^ **value** |
| --- | --- | --- | --- | --- | --- |
| ACC | Normal | 128 | 1.17 | 0.598 | 0.007 |
|  | Tumor | 77 | 0.88 | 0.803 |  |
| BLCA | Normal | 28 | 0.93 | 0.858 | 0.432 |
|  | Tumor | 407 | 0.864 | 0.741 |  |
| BRCA | Normal | 292 | 1.884 | 0.96 | 0.004 |
|  | Tumor | 1099 | 1.74 | 0.988 |  |
| CESC | Normal | 13 | 1.245 | 0.502 | 0.156 |
|  | Tumor | 306 | 0.96 | 0.694 |  |
| CHOL | Normal | 9 | 0.766 | 0.308 | 0.003 |
|  | Tumor | 36 | 1.032 | 0.598 |  |
| COAD | Normal | 349 | 0.864 | 0.582 | 0.000 |
|  | Tumor | 290 | 0.614 | 0.541 |  |
| DLBC | Normal | 444 | 0.233 | 0.458 | 0.000 |
|  | Tumor | 47 | 0.731 | 0.435 |  |
| ESCA | Normal | 666 | 0.856 | 0.528 | 0.645 |
|  | Tumor | 182 | 0.82 | 0.744 |  |
| GBM | Normal | 1157 | 0.956 | 0.528 | 0.000 |
|  | Tumor | 166 | 2.804 | 0.841 |  |
| HNSC | Normal | 44 | 0.673 | 0.67 | 0.000 |
|  | Tumor | 520 | 0.575 | 0.63 |  |
| KICH | Normal | 53 | 1.895 | 1.585 | 0.002 |
|  | Tumor | 66 | 1.331 | 0.599 |  |
| KIRC | Normal | 100 | 2.058 | 0.896 | 0.000 |
|  | Tumor | 531 | 1.718 | 0.791 |  |
| KIRP | Normal | 60 | 1.63 | 0.975 | 0.735 |
|  | Tumor | 289 | 1.692 | 1.031 |  |
| LAML | Normal | 70 | 0.345 | 0.245 | 0.000 |
|  | Tumor | 173 | 1.692 | 0.896 |  |
| LGG | Normal | 1152 | 0.952 | 0.528 | 0.000 |
|  | Tumor | 523 | 2.454 | 0.811 |  |
| LIHC | Normal | 160 | 0.761 | 0.488 | 0.141 |
|  | Tumor | 371 | 0.678 | 0.553 |  |
| LUAD | Normal | 347 | 1.233 | 0.84 | 0.793 |
|  | Tumor | 515 | 1.176 | 0.799 |  |
| LUSC | Normal | 338 | 1.18 | 0.822 | 0.009 |
|  | Tumor | 498 | 1.036 | 0.803 |  |
| MESO | Tumor | 87 | 0.978 | 0.734 |  |
| OV | Normal | 88 | 1.795 | 0.874 | 0.000 |
|  | Tumor | 427 | 2.108 | 1.064 |  |
| PAAD | Normal | 171 | 1.043 | 0.611 | 0.203 |
|  | Tumor | 179 | 1.091 | 0.639 |  |
| PCPG | Normal | 3 | 0.623 | 0.44 | 0.543 |
|  | Tumor | 182 | 0.465 | 0.345 |  |
| PRAD | Normal | 152 | 1.428 | 0.744 | 0.041 |
|  | Tumor | 496 | 1.333 | 0.584 |  |
| READ | Normal | 318 | 0.864 | 0.604 | 0.003 |
|  | Tumor | 93 | 0.678 | 0.476 |  |
| SARC | Normal | 2 | 1.074 | 0.396 | ns |
|  | Tumor | 262 | 1.272 | 0.938 |  |
| SKCM | Normal | 813 | 1.328 | 0.788 | 0.000 |
|  | Tumor | 469 | 1.014 | 0.849 |  |
| STAD | Normal | 210 | 0.868 | 0.499 | 0.222 |
|  | Tumor | 414 | 0.811 | 0.601 |  |
| TGCT | Normal | 165 | 2.186 | 0.393 | 0.000 |
|  | Tumor | 154 | 1.192 | 0.892 |  |
| THCA | Normal | 338 | 1.575 | 0.894 | 0.000 |
|  | Tumor | 512 | 1.144 | 0.658 |  |
| THYM | Normal | 446 | 0.239 | 0.458 | 0.000 |
|  | Tumor | 119 | 1.614 | 0.779 |  |
| UCEC | Normal | 101 | 1.66 | 0.632 | 0.679 |
|  | Tumor | 181 | 1.655 | 1.094 |  |
| UCS | Normal | 78 | 1.616 | 0.776 | 0.001 |
|  | Tumor | 57 | 1.007 | 1.129 |  |
| UVM | Tumor | 79 | 0.782 | 0.501 |  |

* Wilcoxon rank sum test

ACC, adrenal cortical carcinoma; BLCA, bladder urothelial carcinoma; BRCA, breast invasive carcinoma; CESC, Cervical squamous cell carcinoma and endocervical adenocarcinoma; CHOL, cholangiocarcinoma; COAD, colon adenocarcinoma; DLBC, lymphoid neoplasm diffuse large b-cell lymphoma; ESCA, esophageal carcinoma; GBM, glioblastoma; HNSC, head and neck squamous cell carcinoma; KICH, kidney chromophobe; KIRC, kidney renal clear cell carcinoma; KIRP, kidney renal papillary cell carcinoma; LAML, acute myeloid leukemia; LGG, lower grade glioma; LIHC, liver hepatocellular carcinoma; LUAD, lung adenocarcinoma; LUSC, lung squamous cell carcinoma; MESO, mesothelioma; OV, ovarian serous cystadenocarcinoma; PAAD, pancreatic adenocarcinoma; PCPG, pheochromocytoma and paraganglioma; PRAD, prostate adenocarcinoma; READ, rectum adenocarcinoma; SARC, sarcoma; SKCM, skin cutaneous melanoma; STAD, stomach adenocarcinoma; TGCT, testicular germ cell tumors; THCA, thyroid carcinoma; THYM, thymoma; UCEC, uterine corpus endometrial carcinoma; UCS, uterine carcinosarcoma; UVM, uveal melanoma

Supplementary Table S4 Associations between progression-free interval and clinicopathological characteristics in patients in TCGA using Cox regression

| **Characteristics** | **Total (N)** | **HR (95% CI) Univariate analysis** | ***P* value Univariate analysis** | **HR (95% CI) Multivariate analysis** | ***P* value Multivariate analysis** |
| --- | --- | --- | --- | --- | --- |
| Age (<=60 vs. >60) | 669 | 0.341(0.267-0.435) | <0.001 | 0.603(0.394-0.921) | 0.019 |
| Gender (Female vs. Male) | 669 | 0.936(0.749-1.171) | 0.564 |  |  |
| Race (Asian vs. Black or African American & White) | 657 | 0.748(0.240-2.336) | 0.618 |  |  |
| Primary therapy outcome (CR vs. PD & SD & PR) | 443 | 0.311(0.199-0.487) | <0.001 | 0.339(0.210-0.549) | <0.001 |
| WHO grade (G2 vs. G3 & G4) | 612 | 0.361(0.274-0.476) | <0.001 | 0.869(0.606-1.245) | 0.443 |
| Histological type (Astrocytoma & Oligoastrocytoma & Oligodendroglioma vs. Glioblastoma) | 669 | 0.169(0.133-0.214) | <0.001 | 0.536(0.162-1.780) | 0.309 |
| IDH status (WT vs. Mut) | 660 | 7.127(5.562-9.131) | <0.001 | 3.943(2.490-6.243) | <0.001 |
| 1p/19q codeletion (Codel vs. Non-codel) | 663 | 0.293(0.209-0.410) | <0.001 | 0.793(0.517-1.217) | 0.289 |
| EGFR status (WT vs. Mut) | 655 | 0.269(0.200-0.362) | <0.001 | 0.508(0.278-0.926) | 0.027 |
| PIK3CA status (WT vs. Mut) | 655 | 0.730(0.491-1.085) | 0.119 |  |  |
| WEE2-AS1 (High vs. Low) | 669 | 1.825(1.458-2.283) | <0.001 | 1.424(1.018-1.990) | 0.039 |

Supplementary Table S5 Associations between disease-specific survival and clinicopathological characteristics in patients in TCGA using Cox regression

| **Characteristics** | **Total (N)** | **HR (95% CI)**  **Univariate**  **analysis** | **P value**  **Univariate analysis** | **HR (95% CI) Multivariate analysis** | **P value Multivariate analysis** |
| --- | --- | --- | --- | --- | --- |
| Age (<=60 vs. >60) | 648 | 0.217(0.163-0.288) | <0.001 | 0.286(0.170-0.482) | <0.001 |
| Gender (Female vs. Male) | 648 | 0.814(0.622-1.064) | 0.132 |  |  |
| Race (Asian vs. Black or African American & White) | 637 | 0.605(0.150-2.435) | 0.479 |  |  |
| Primary therapy outcome (CR vs. PD & SD & PR) | 439 | 0.218(0.101-0.470) | <0.001 | 0.245(0.104-0.574) | 0.001 |
| WHO grade (G2 vs. G3 & G4) | 592 | 0.162(0.108-0.245) | <0.001 | 0.483(0.288-0.812) | 0.006 |
| Histological type (Astrocytoma & Oligoastrocytoma & Oligodendroglioma vs. Glioblastoma) | 648 | 0.110(0.083-0.147) | <0.001 | 0.344(0.100-1.190) | 0.092 |
| IDH status (WT vs. Mut) | 639 | 10.413(7.716-14.054) | <0.001 | 3.544(1.975-6.359) | <0.001 |
| 1p/19q codeletion (Codel vs. Non-codel) | 643 | 0.200(0.123-0.324) | <0.001 | 0.842(0.448-1.580) | 0.592 |
| EGFR status (WT vs. Mut) | 634 | 0.279(0.202-0.386) | <0.001 | 0.696(0.330-1.468) | 0.341 |
| PIK3CA status (WT vs. Mut) | 634 | 0.966(0.588-1.587) | 0.891 |  |  |
| WEE2-AS1 (High vs. Low) | 648 | 2.100(1.600-2.756) | <0.001 | 2.051(1.288-3.265) | 0.002 |
